# The Weimberg pathway: an alternative for *Myceliophthora thermophila* to utilize d-xylose

**DOI:** 10.1186/s13068-023-02266-7

**Published:** 2023-01-23

**Authors:** Defei Liu, Yongli Zhang, Jingen Li, Wenliang Sun, Yonghong Yao, Chaoguang Tian

**Affiliations:** 1 Key Laboratory of Engineering Biology for Low-carbon Manufacturing, Tianjin Institute of Industrial Biotechnology, Chinese Academy of Sciences, Tianjin, 300308 China; 2 National Technology Innovation Center of Synthetic Biology, Tianjin, 300308 China

**Keywords:** *Myceliophthora*, d-Xylose, d-Xylose dehydrogenase, Weimberg pathway, 1,2,4‐Butanetriol

## Abstract

**Background:**

With d-xylose being the second most abundant sugar in nature, its conversion into products could significantly improve biomass-based process economy. There are two well-studied phosphorylative pathways for d-xylose metabolism. One is isomerase pathway mainly found in bacteria, and the other one is *oxo*-reductive pathway that always exists in fungi. Except for these two pathways, there are also non-phosphorylative pathways named xylose oxidative pathways and they have several advantages over traditional phosphorylative pathways. In *Myceliophthora thermophila*, d-xylose can be metabolized through *oxo*-reductive pathway after plant biomass degradation. The survey of non-phosphorylative pathways in this filamentous fungus will offer a potential way for carbon-efficient production of fuels and chemicals using d-xylose.

**Results:**

In this study, an alternative for utilization of d-xylose, the non-phosphorylative Weimberg pathway was established in *M. thermophila*. Growth on d-xylose of strains whose d-xylose reductase gene was disrupted, was restored after overexpression of the entire Weimberg pathway. During the construction, a native d-xylose dehydrogenase with highest activity in *M. thermophila* was discovered. Here, *M. thermophila* was also engineered to produce 1,2,4‐butanetriol using d-xylose through non-phosphorylative pathway. Afterwards, transcriptome analysis revealed that the d-xylose dehydrogenase gene was obviously upregulated after deletion of d-xylose reductase gene when cultured in a d-xylose medium. Besides, genes involved in growth were enriched in strains containing the Weimberg pathway.

**Conclusions:**

The Weimberg pathway was established in *M. thermophila* to support its growth with d-xylose being the sole carbon source. Besides, *M. thermophila* was engineered to produce 1,2,4‐butanetriol using d-xylose through non-phosphorylative pathway. To our knowledge, this is the first report of non-phosphorylative pathway recombinant in filamentous fungi, which shows great potential to convert d-xylose to valuable chemicals.

**Supplementary Information:**

The online version contains supplementary material available at 10.1186/s13068-023-02266-7.

## Background

**Fig. 1:**
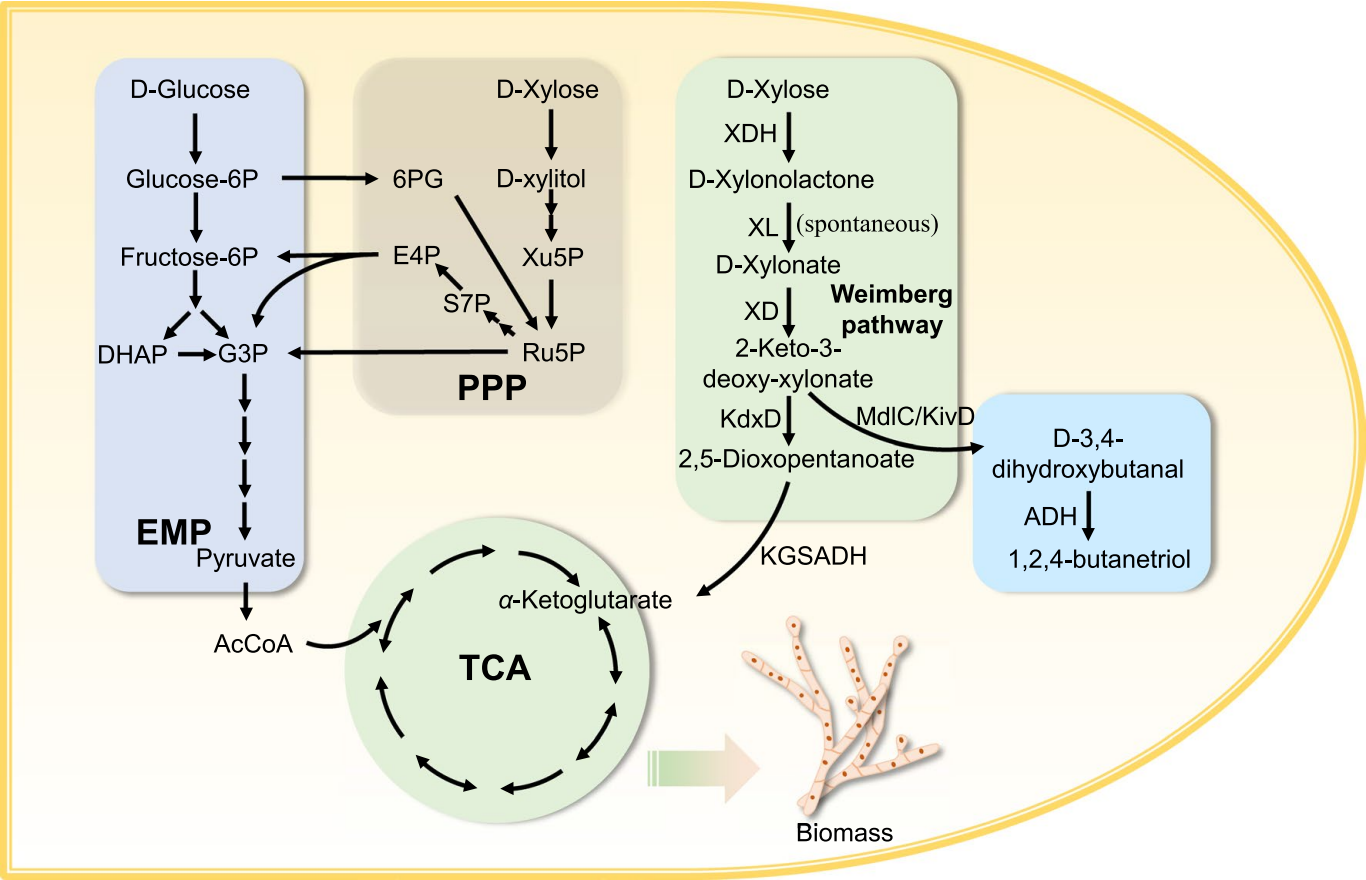
Map of central carbon metabolism of *M. thermophila*, the Weimberg pathway and BT biosynthetic pathway. Abbreviations: XDH d-xylose dehydrogenase; XL d-xylonolactonase; XD d-xylonate dehydratase; KdxD 2-keto-3-deoxy-d-xylonate dehydratase; KGSADH 2-ketoglutarate semialdehyde dehydrogenase; MdlC benzoylformate decarboxylase; KviD 2-ketoisovalerate decarboxylase; EMP Embden–Meyerhof–Parnas pathway; PPP, pentose phosphate pathway; TCA tricarboxylic acid cycle; DHAP dihydroxyacetone phosphate; G3P glyceraldehyde 3-phosphate, 6PG 6-phospho-d-gluconate; E4P erythrose 4-phosphate; S7P sedoheptulose 7-phosphate; Xu5P xylulose-5P; Ru5P ribulose-5P; AcCoA acetyl-coenzyme A

Lignocellulosic biomass has attracted much attention as a promising renewable resource for production of biofuels and bioproducts. In lignocellulose hydrolysis, d-xylose is the predominant pentose with fraction varying from 5 to 35% [1]. Therefore, it is very important to explore utilization and bioconversion of d-xylose. There are two well-studied phosphorylative pathways for d-xylose metabolism, which are isomerase pathway and *oxo*-reductive pathway. The isomerase pathway is mainly found in bacteria, where d-xylose is isomerized to d-xylulose, and then phosphorylated into d-xylulose-5-phosphate. While the *oxo*-reductive pathway is more common in fungi, where d-xylose is reduced to d-xylitol, and then oxidized to d-xylulose and phosphorylated to d-xylulose-5-phosphate (Fig. 1). d-Xylulose-5-phosphate produced by these two pathways was further utilized through pentose phosphate pathway (PPP) for cellular metabolite synthesis [2].

Except for these two well-known phosphorylative pathways for d-xylose metabolism as mentioned above, there are also non-phosphorylative pathways named xylose oxidative pathways (XOPs). In these pathways, d-xylose is first dehydrogenized into d-xylonolactone by d-xylose dehydrogenase (XDH). Then, d-xylonolactone is hydrolyzed into d-xylonate by d-xylonolactonase (XL) or spontaneously. d-Xylonate is subsequently dehydrated to 2-keto-3-deoxy-d-xylonate by d-xylonate dehydratase (XD) (Fig. 1). Thereafter, XOPs branch off to produce pyruvate and glycolaldehyde (route I or Dahms pathway) [3], or α-ketoglutarate (route II or Weimberg pathway) [4], or pyruvate and glycolate (route III) [5]. Entire or partial genes of these pathways are found in most archaea, some bacteria and fungi [2]. XOPs have several advantages compared with traditional phosphorylative pathways. First of all, XOPs have fewer enzymatic reaction steps when forming key intermediates such as α-ketoglutarate, pyruvate and glycolate. Also, Weimberg pathway, one of XOPs, provides a more carbon-efficient route for xylose-derived α-ketoglutarate production. Another advantage of XOPs is their independence from other carbohydrate assimilative pathways. Hence, great potential lies in pathways that are designed to utilize five-carbon and six-carbon sugar simultaneously or orthogonally with XOPs [6–8]. Furthermore, when comparing with phosphorylative pathways, XOPs show thermodynamic favorability due to their highly negative *ΔrG* values in reactions [2].

Previously, the upper part of XOPs has been established in *Kluyveromyces lactis* [9], *Saccharomyces cerevisiae* [10], and *Escherichia coli* [11] to convert xylose to xylonate. Further, the entire Weimberg pathway was constructed in *E. coli* [12, 13], *S. cerevisiae* [14] and *Corynebacterium glutamicum* [15, 16], enabling them to grow on d-xylose as sole carbon source. Besides, XOPs were also employed to produce chemicals, such as 1,4-butanediol [13] and 1,2,4‐butanetriol (BT) [17, 18].

*M. thermophila* is a thermophilic ascomycete fungus that can efficiently degrade and utilize various carbohydrates from plant biomass [19]. Currently, this fungus has been engineered to become a cell factory to produce enzymes [20, 21] and various commodity chemicals [22–25]. Therefore, it is very important to explore xylose consumption routes without carbon loss in *M. thermophila* in order to generate lignocellulose-derived products from plant biomass efficiently. In this study, a functional native XDH encoding gene *Mtxyd1* was discovered. Interestingly, *Mtxyd1* was induced by d-xylose when d-xylose reductase gene was disabled. The entire Weimberg pathway was established in *M. thermophila*, enabling it to generate biomass using d-xylose as sole carbon source. Besides, XOP was employed to produce BT from d-xylose in *M. thermophila*.

## Results

### Identification of xylose reductase gene in *M. thermophila*

**Fig. 2:**
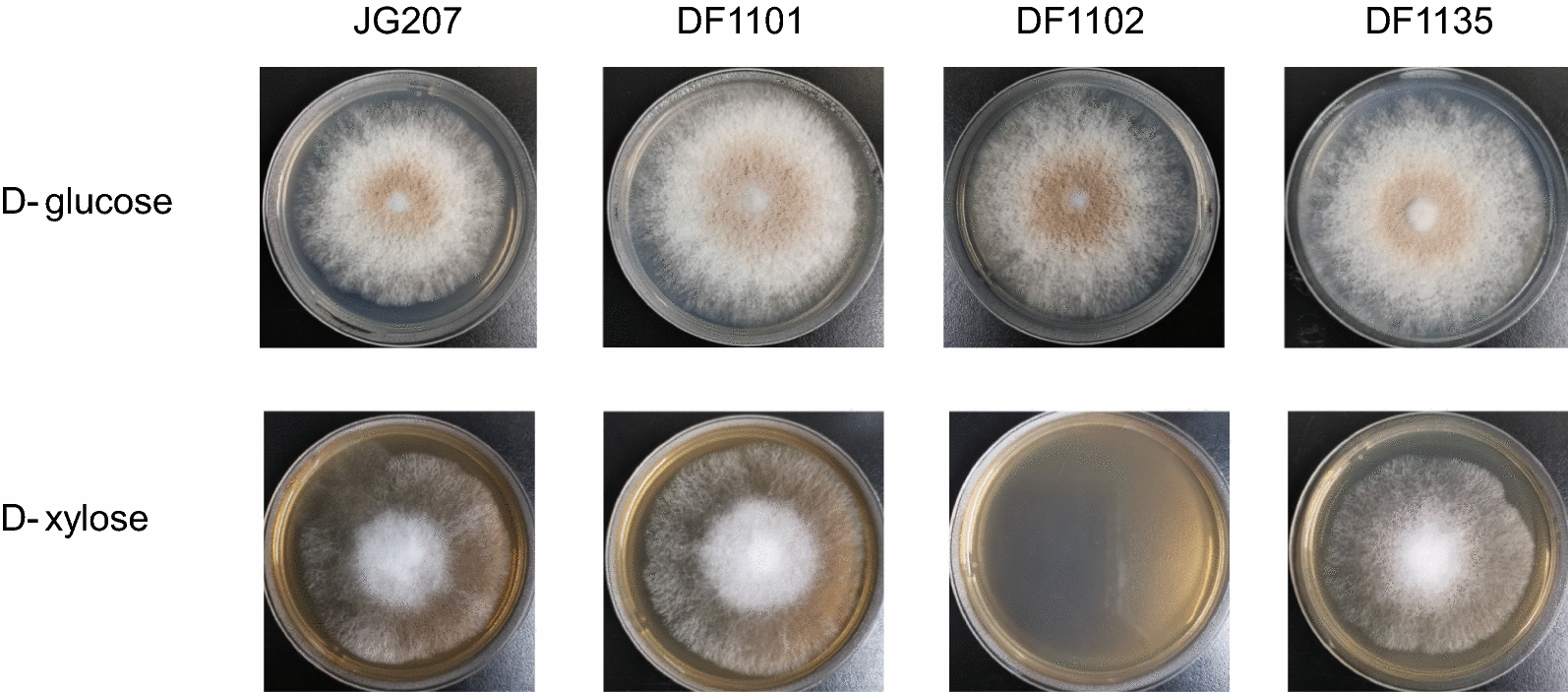
Growth phenotype of JG207, deletion and complementation mutants on GVMM and XVMM agar plates

Belonging to aldo–keto reductase family, xylose reductase catalyzes the first step of xylose assimilation in fungi by converting xylose into xylitol. In order to test the Weimberg pathway function and prevent xylose assimilation through *oxo*-reductive route, xylose reductase was identified in *M*. *thermophila* by deletion and complementation of target genes. Two genes (*MYCTH_43671* and *MYCTH_113236*) were annotated as d-xylose reductase in genome-scale metabolic model (GEM) of *M. thermophila* [26]. Hence, these two genes that belong to aldo–keto reductase family were tested. Results (Fig. 2) showed that xylose utilization was hindered after deletion of *MYCTH_43671* (strain DF1102). While there were no obvious effects after deletion of *MYCTH_113236* (strain DF1101). Growth phenotype was restored as it was in JG207 after complementation of *MYCTH_43671* (strain DF1135). Given the test results and homology (84.09% identity) with xylose reductase gene (*NCU08384*) in *Neurospora crassa* OR74A, *MYCTH_43671* (*Mtxyl1*) was identified as the coding gene of xylose reductase in *M. thermophila*.

### Expression of Weimberg pathway in *M. thermophila*

**Fig. 3:**
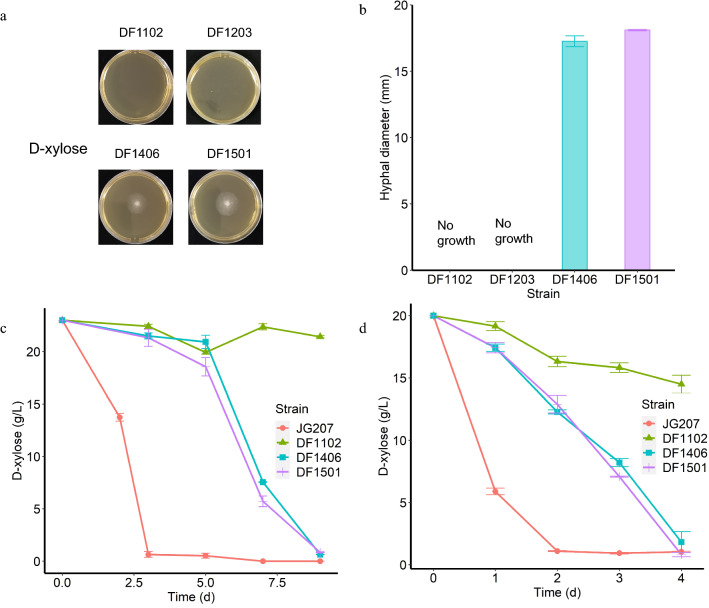
Growth phenotype (**a**) and hyphal diameter (**b**) of strains engineered with the Weimberg pathway genes on XVMM agar plates; d-xylose consumption of strains inoculated with spores (**c**) and 2 g (wet weight) mycelium (**d**)

The Weimberg pathway from *C. crescentus* was integrated into genome of *M. thermophila* through two rounds of transformation using the CRISPR/Cas9 system [27]. The first two genes *xylB* and *xylC* were inserted in *pck* (*MYCTH_2315623*) locus to obtain DF1230. Then, the lower part of Weimberg pathway which consist of *xylD*, *xylX*, and *xylA* was integrated in *bar* locus after the first two steps. *Mtxyl1* was also deleted at the same time to get strain DF1203 that contained all Weimberg pathway genes. Besides, DF1232 was obtained after deletion of *Mtxyl1* in DF1230. Growth phenotype results showed that DF1102 and DF1203 could not grow on 1 × Vogel’s minimal medium (VMM) plates with d-xylose as sole carbon source (Fig. 3a). In order to further explore d-xylose consumption, liquid fermentation in shake flask was tested. Unsurprisingly, it’s hard for DF1102 and DF1203 to germinate after inoculating spores into VMM supplemented with 2% (w/v) d-xylose (XVMM) in 6 days. Therefore, wet mycelium transfer was used for d-xylose consumption test. Results revealed that d-xylose was utilized very slowly by DF1102, DF1232, and DF1203 in mycelium transfer. Compared with DF1102 and DF1232, d-xylose consumption of DF1203 was not significantly improved (Additional file 1: Fig. S1a).

### Exploring higher activity of XDH

XDH is the first step of Weimberg pathway to convert d-xylose to d-xylonate. The accumulation of d-xylonate was inhibitory to *S. cerevisiae* due to the decrease of intracellular pH that may lead to cell death consequently [28, 29]. However, this phenomenon did not happen in *M. thermophila* mutant that contained *xylB* and *xylC* when cultured in VMM plate supplied with d-xylose (Additional file 2: Fig. S2). Furthermore, no obvious d-xylonate peaks were observed in intracellular and extracellular metabolites of DF1232 and DF1203 using HPLC (Additional file 1: Fig. S1b, c). Hence, XDH may not function well when coded by *xylB*.

**Fig. 4:**
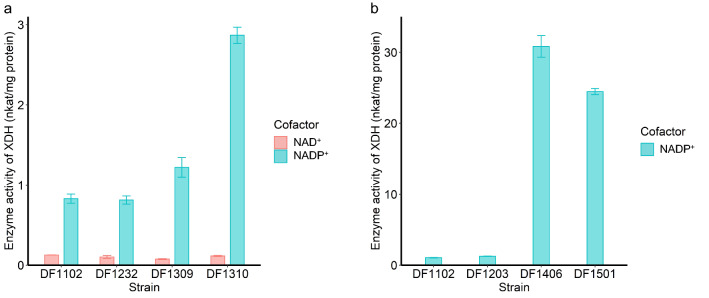
Specific activity of d-xylose dehydrogenase in different recombinant *M. thermophila* strains (**a**: DF1102, DF1232, DF1309 and DF1310; **b**: DF1102, DF 1203, DF1406 and DF1501)

As the first step of Weimberg pathway, XDH activity was mainly observed in prokaryotes [2]. Aside from prokaryotes, there are also reports about XDH activity in eukaryotic microorganisms [30, 31]. Therefore, another XDH coding gene *Trxyd1* (*TRIREDRAFT_53673*) was cloned from cDNA of *T. reesei* QM6a. In addition, an endogenous *Mtxyd1* (*MYCTH_112471*, 69.70% identity with *Trxyd1*), which was annotated as XDH gene, was also cloned. These two new XDH sequences were inserted into *M. thermophila* genome. The activities of XDHs were tested in different recombinant *M. thermophila* strains with NAD^+^ and NADP^+^ as cofactors, respectively (Fig. 4a). Results showed that XDH encoded by *xylB* presented extremely low activity. This was similar to the activity of control strain DF1102 when added with NAD^+^ or NADP^+^. When using NADP^+^ as a cofactor, XDH encoded by endogenous *Mtxyd1* owned the highest activity, which was 2.30 and 3.60 times higher than xylB and Trxyd1. In conclusion, XDH encoded by *Mtxyd1* represented good compatibility with this thermophilic fungus.

The enzymatic activity of XDH in strains DF1309 and DF1310 was verified, but the accumulation of d-xylonate still could not be examined in extracellular metabolites using high-performance liquid chromatography (HPLC) (Additional file 3: Fig. S3a). Thus, a more sensitive method gas chromatography–mass spectrometry (GC–MS) was used to detect d-xylonate. Results revealed that d-xylonate could be detected in extracellular metabolites of all tested strains, even in DF1102 (Additional file 3: Fig. S3b). Transcriptome analysis indicated that *Mtxyd1* was obviously upregulated in DF1102 when cultured in d-xylose media (see below), revealing the reason why d-xylonate could be tested in DF1102.

### Overexpressing the Weimberg pathway genes in ***M. thermophila ***Δ***Mtxyl1*** mutant restored its growth on d-xylose

After knocking in the *C. crescentus* Weimberg pathway, *M. thermophila* still could not grow on VMM plate when supplied only with d-xylose. Low activity of XDH encoded by *xylB* might be the main reason. Previous reports showed that the conversion of d-xylonolactone into d-xylonate occurred spontaneously at neutral pH [28, 29]. Hence, only *Mtxyd1* was selected in following construction of the Weimberg pathway in *M. thermophila* to convert d-xylose to d-xylonate. In addition, multicopy of the lower Weimberg pathway enabled yeast biomass assimilation [14]. Thus, strong constitutive promoters *Ptef* and *Pgpda* were used for *Mtxyd1*and *xylD*, respectively. Then, a new Weimberg pathway gene group including *Mtxyd1*, *xylD*, *xylX*, and *xylA* was randomly inserted into the genome of *M. thermophila* to obtain DF1406. Besides, it was reported that KGSADH encoded by *ksaD* from *C. glutamicum* functioned better than KGSADH encoded by *xylA* from *C. crescentus* when expressed in *S. cerevisiae* [14]. Moreover, k*cat* of 2-keto-3-deoxy-d-xylonate dehydratase (KdxD) (*BxxylX*) from *Burkholderia xenovorans* was ninefold higher than that of KdxD (*xylX*) from *C. crescentus* [13]. Therefore, another mutant DF1501 that contained *Mtxyd1*, *xylD*, *BxxylX* and *ksad* was constructed using the same way as DF1406.

The gene number showed that multicopy of Weimberg pathway genes were inserted into the genome of *M. thermophila* (Additional file 4: Fig. S4a). Growth of DF1406 and DF1501 on XVMM plate was restored (Fig. 3a). d-Xylose consumption of these two strains were also measured in flask. Results showed that strains containing Weimberg pathway could germinate and utilize d-xylose in liquid medium when inoculated with spores (Fig. 3c). Besides, d-xylose consumption of DF1406 and DF1501 was improved to 4.60 g/L/day and 5.17 g/L/day, which was about 3 times higher than DF1102, when cultured with mycelium transfer (Fig. 3d). Moreover, there is 1.90 and 3.19 times increase of d-xylonate and α-ketoglutarate, respectively, in intracellular metabolites of DF1501 compared with DF1102 (Additional file 5: Fig. S5a, b). The activity of XDH in DF1406 and DF1501 was about ten times higher than that in DF1310 which contained one copy of *Mtxyd1* (Fig. 4). The lower part activity of the pathway that included XylD, XylX/BxXylX and XylA/KsaD was assessed through the combined enzyme activity by determining NADH formation from d-xylonate. However, no obvious enzymatic activity was detected (Additional file 6: Fig. S6). This suggested that the activity of lower part of the pathway was probably the limit step to form biomass via Weimberg pathway.

### Engineering *M. thermophila* to produce BT using XOP

**Fig. 5:**
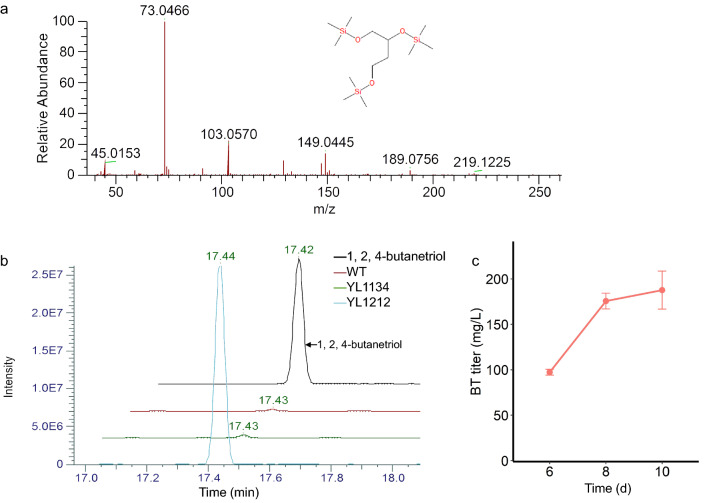
**a** MS spectra of BT at 17.42 min. **b** GC–MS analysis of extracellular metabolites of WT, YL1134 and YL1212 at day 8. **c** BT titer of YL1212

XOPs have already been employed for chemical synthesis from lignocellulosic biomass in many microorganisms, such as *E. coli* [13], *Saccharomyces cerevisiae* [18]. Filamentous fungi offer great potential in using lignocellulosic biomass to synthesize chemicals. Synthesis steps of BT from d-xylose share three upper enzymes with the Weimberg pathway. Thus, engineering *M. thermophila* to produce BT was explored with wild-type *M. thermophila* ATCC42464 (WT) as starting strain. Firstly, the gene set of *Mtxyd1*, *xylC*, *xylD*, *kivD*, and *adh1* was overexpressed in *M. thermophila* to obtain strain YL1134 in just one round of transformation. Production of BT was not detected via GC–MS after measuring extracellular metabolites of YL1134 at day 8 (Fig. 5b). In *E. coli*, the activity of benzoylformate decarboxylase is the key limit factor for BT production [32]. Therefore, another candidate gene of benzoylformate decarboxylase *mdlC* from *Pseudomonas putida* was overexpressed in YL1134 to get YL1212. After 10 days of fermentation, production of BT was verified by GC–MS (Fig. 5a, b) and the titer of BT reached 187.7 mg/L (Fig. 5c).

### Transcriptional profile of *M. thermophila* with the Weimberg pathway

**Fig. 6:**
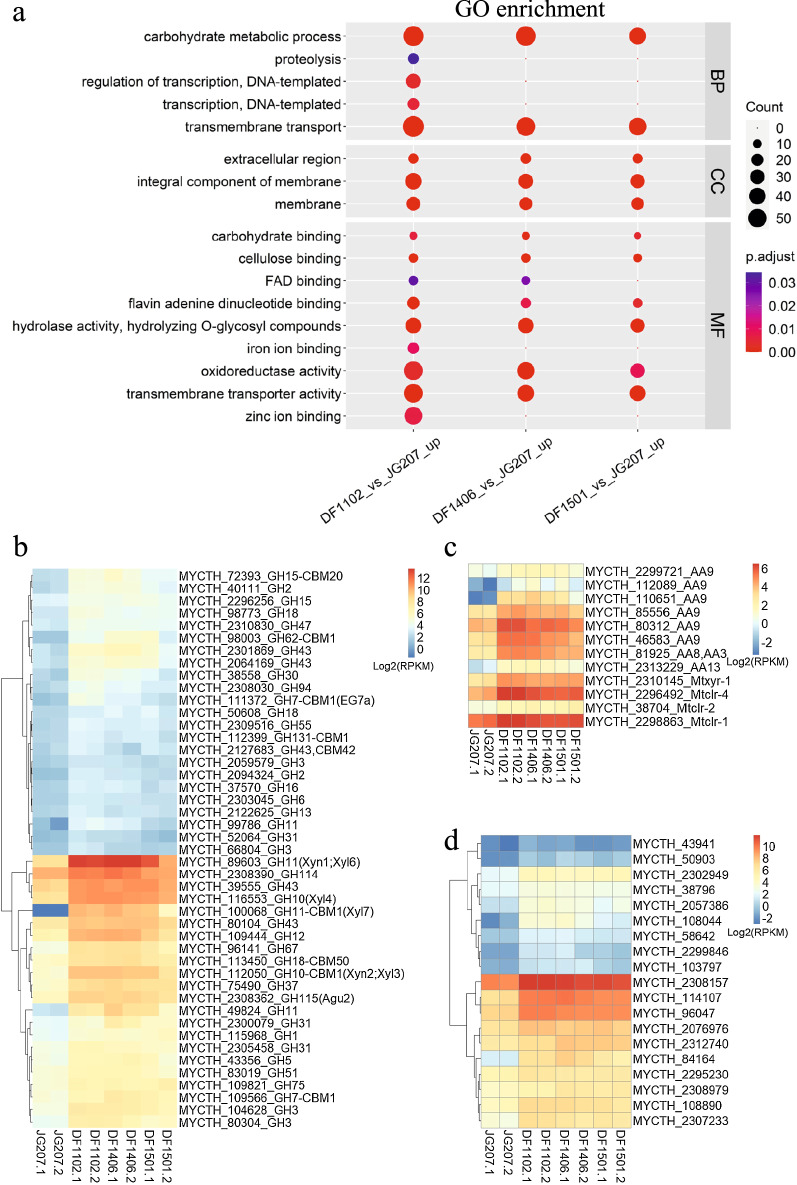
**a** Functional enrichment analysis of upregulated genes in DF1102, DF1406 and DF1501 (fold change ≥ 2, adjusted *p* value ≤ 0.05) compared with JG207. Heatmap of upregulated genes belonging to GH family (**b**), AA family and regulators (**c**), and sugar transporters (**d**)

RNA-seq was used to study the effect of *Mtxyd1* deletion and the Weimberg pathway insertion at transcript level. Starting strain JG207 and its mutants DF1102, DF1406, DF1501 were pre-cultured in VMM supplemented with 2% glucose (GVMM). Then, filtered mycelium was transferred to VMM with 10 g/L d-xylose for 4 h. Compared with JG207, a group of genes was notably enriched in carbohydrate metabolic process in strain DF1102 (Fig. 6a). These genes mainly belonged to GH and AA families (Fig. 6b, c) which were related to carbohydrate-active enzymes (CAZymes). In GH family, genes that encode hydrolytic enzymes (GH10, GH11, GH43) in xylan degradation were remarkably upregulated. Besides, lytic polysaccharide monooxygenases (LPMOs) which were classified in AA9 family were also significantly upregulated. LPMOs were known to boost the hydrolytic breakdown of lignocellulosic biomass due to their oxidative mechanism [33, 34]. Moreover, positive regulators of cellulase and some hemicellulase genes (*clr-1*, *clr-2*, *clr-4*), xylanase and xylose utilization genes (*xyr-1*) were upregulated. In addition, a group of genes which were annotated as sugar transporter also were enriched (Fig. 6a, d). Similarly, most upregulated genes in DF1102 were also increased in strain DF1406 and DF1501 when compared with JG207. After deletion of *Mtxyl1*, the expression of endogenous XDH gene *Mtxyd1* in DF1102 increased by 5 times when induced by d-xylose in comparison with JG207 (Additional file 4: Fig. S4b).

**Fig. 7:**
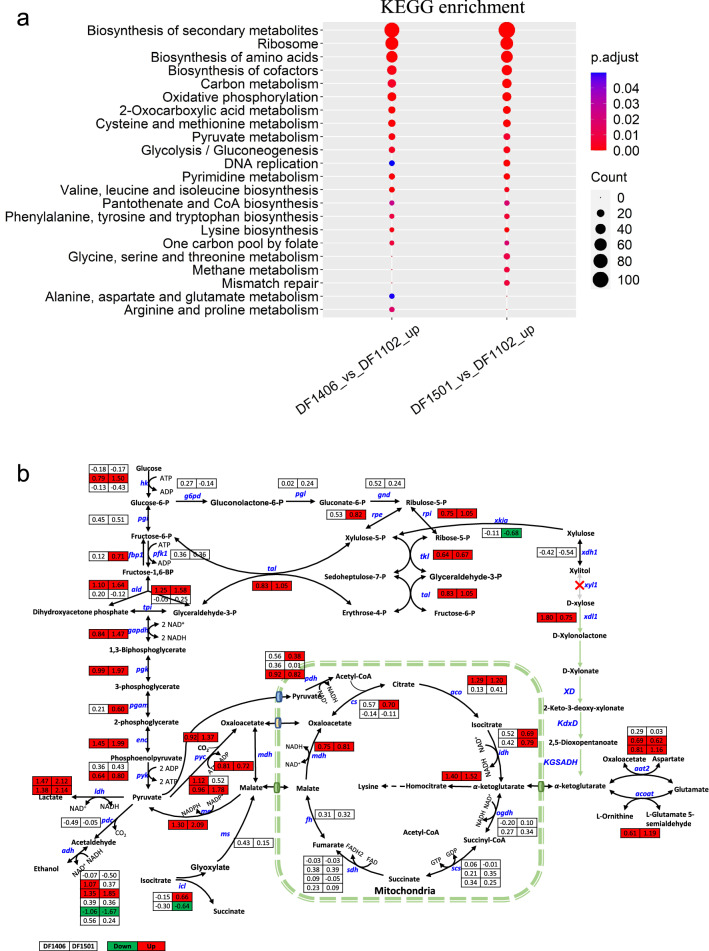
**a** Functional enrichment analysis of upregulated genes in DF1406 and DF1501 (fold change ≥ 1.5, adjusted *p* value ≤ 0.05) compared with DF1102. **b** Expression of genes in central metabolic pathway in engineered *M. thermophila* strain DF1406 and DF1501 compared with DF1102. Numbers near reactions represent log2-fold change in strains carrying the Weimberg pathway compared with DF1102 (upregulated genes (fold change ≥ 1.5, adjusted *p* value ≤ 0.05) are shown in red; downregulated genes (fold change ≤ 1.5, adjusted *p* value ≤ 0.05) are shown in green)

The insertion of Weimberg pathway not only increased the consumption of d-xylose, but also affected its metabolism profiles at transcript level. Compared with DF1102, upregulated genes in DF1406 and DF1501 possessed similar KEGG enrichment in metabolic process. Increased genes were mainly enriched in biosynthesis of secondary metabolites, ribosome, biosynthesis of amino acids, biosynthesis of cofactors and carbon metabolism (Fig. 7a), all of which were believed to be important for cell growth. Besides, gene expression was mapped onto central carbon metabolism (Fig. 7b). Genes in glycolysis, PPP, and part of tricarboxylic acid cycle (TCA) were greatly upregulated to support strain growth when using the Weimberg pathway.

### Metabolic profile of *M. thermophila* with the Weimberg pathway predicted by GEM

**Fig. 8:**
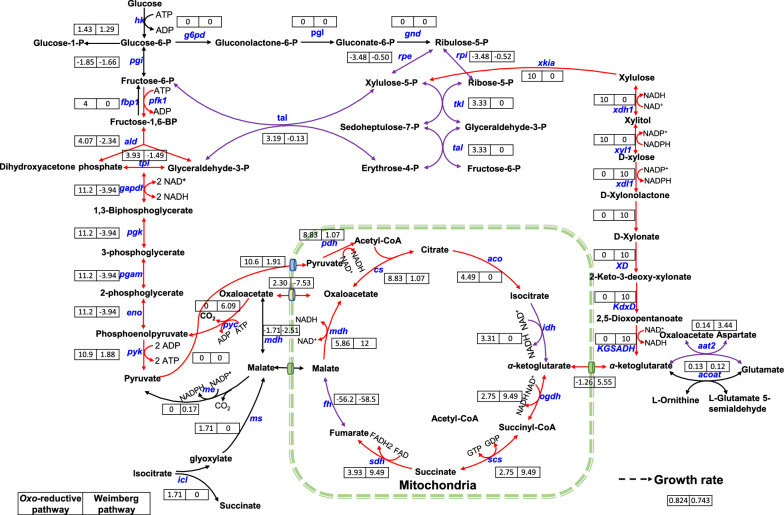
The flux distribution map predicted by GEM of *M. thermophila* iDL1450. Numbers near reactions represent fluxes in *oxo*-reduction and the Weimberg pathway. Color of reaction arrows reflect the flux differences between *oxo*-reduction and the Weimberg pathway (red: flux difference ≥ 4, purple: 4 > flux difference ≥ 2, black: flux difference < 2)

Generally, α-ketoglutarate which is produced from the Weimberg pathway participates in TCA cycle to produce energy for cell growth. Cells also need to biosynthesize other components from intermediates, such as pyruvate, phosphoenolpyruvate and sugars. In strains with the Weimberg pathway, these intermediates are probably produced via other pathways, such as gluconeogenesis. In order to explore the metabolic profile in starting stain and its mutants, flux distributions were predicted using GEM of *M. thermophila* iDL1450 [26] with flux balance analysis (FBA) [35] (Fig. 8, Additional file 7). Results showed that 64.7% flux of phosphoenolpyruvate was utilized in gluconeogenesis in strains which contained the Weimberg pathway. ATP synthesis rate was also calculated using d-xylose as sole carbon source. The Weimberg pathway generated 26.6 mol ATP/mol d-xylose, whereas *oxo*-reduction pathway generated 28.3 mol ATP/mol d-xylose via PPP. At the same d-xylose uptake rate, growth rate in Weimberg pathway was lower than *oxo*-reduction pathway. The different flux distributions and low ATP synthesis rate of the Weimberg pathway probably caused lower growth efficiency compared with *oxo*-reduction pathway. The measured growth rate of DF1501 with the Weimberg pathway was 66.7% lower than the starting strain using *oxo*-reduction pathway (Additional file 8: Fig. S7).

## Discussion

As one of the main sugars in lignocellulose, d-xylose can be utilized through *oxo*-reduction pathway in fungi where d-xylose is reduced to xylitol by a d-xylose reductase in the first place [36]. d-Xylose reductase gene in *M. thermophila* was identified by gene homologous search and *Mtxyl1* deletion and complementation experiments. Fungi have complex regulation networks to ferment sugars from lignocellulose. In *M. thermophila*, a number of enzymes and transcriptional regulators involved in plant biomass utilization were reported, such as CRE-1 [37], AmyR [38], CLR-4 [39] and XlnR/Xyr1 [40, 41]. Here, genes related to hemicellulose and xylan-degrading enzymes that yield d-xylose was obviously upregulated after deletion of *Mtxyl1*. Besides, four positive regulator genes of cellulase and xylanase were also upregulated. The deletion of *Mtxyl1* caused some kind of metabolic disorders. Although enough d-xylose was offered in medium, the strain without *Mtxyl1* still tried to upregulate upstream degrading enzymes and d-xylose transporters because the *oxo*-reductive pathway was blocked and low production of downstream metabolites of d-xylose was caused accordingly.

Except for *oxo*-reductive pathway, d-xylose can also be metabolized via XOPs, including the Dahms and Weimberg pathway [2]. XOPs are mainly found in archaea. Some enzymes of XOPs, such as XDH, can also be found in bacterial and fungal species [2]. Different genes that coded XDH were expressed in *M. thermophila.* Unlike yeast [28], XDH coding gene xylB originated from *C. crescentus* did not function well in this filamentous fungus. Thus, another two XDH coding genes, *Trxyd1* and native *Mtxyd1*, from filamentous fungi were tested*.* Results indicated that Mtxyd1 had the highest XDH activity in *M. thermophila*. In yeast, high d-xylonate titer was obtained after expression of *xylB* or *xyd1* [9, 10, 28]. Unexpectedly, among extracellular metabolites of DF1309 and DF1310, d-xylonate was not notably accumulated though obvious XDH activities were detected. Therefore, it is assumed that *M. thermophila* may contain certain regulation mechanism to control d-xylonate concentration to protect cells as *Mtxyd1* is a native coding XDH. Here, the expression of *Mtxyd1* was remarkably upregulated after deletion of *Mtxyl1*. The strain restarted the first step of XOP when *oxo*-reductive pathway was disabled. Here, *Mtxyd1* looks like an evolutionary residue or xylose utilization backup. The toxicity of d-xylonate and low efficiency of XOP may cause microorganisms to abandon this pathway in long-term evolution. While *Mtxyd1* still has an important role in d-xylose utilization because of its positive response to d-xylose. Although the role of XDH in filamentous fungi was still unclear, the results here may offer some clues to explore its function.

In this study, the entire Weimberg pathway was optimized and constructed in thermophilic fungus *M. thermophila*. After overexpression of the entire Weimberg pathway, strain growth of DF1406 and DF1501 on d-xylose was restored with disruption of *Mtxyl1*. d-xylose consumption of the two strains was significantly improved compared with DF1102. Transcriptomic analysis revealed that genes related to cell growth were obviously upregulated in strains carrying Weimberg pathway. In spite of regained growth on d-xylose, DF1406 and DF1501 still had similar CAZyme regulation patterns with DF1102, but they have fewer genes. Results predicted by GEM iDL1450 illuminated that flux distribution of the Weimberg pathway is totally different from that of *oxo*-reductive pathway. Also, the Weimberg pathway showed lower energy efficiency than *oxo*-reductive pathway, which may help to explain the low growth rate in this study and other researches [12, 13]. In *E. coli*, the growth rate of recombinant strain containing the Weimberg pathway was 59% lower than that of wild-type host strain when using isomerase pathway [12]. These data suggest that the more work need to be done to realize the pathway’s potential benefits in heterologous host. Despite the lower energy efficiency, the Weimberg pathway still presents a promising route to produce valuable TCA-cycle derivatives from d-xylose due to its high carbon efficiency [13, 18, 42, 43]. Besides, it provides a parallel metabolic pathway to co-utilize glucose and xylose from hydrolysate of lignocellulosic biomass [6, 7]. Furthermore, *M. thermophila* was also engineered to produce BT, showing the opportunity to synthesize chemicals directly from lignocellulosic biomass through XOP.

## Conclusions

In present study, an alternative d-xylose utilization pathway was explored in *M. thermophila*. Firstly, the gene that coded native d-xylose reductase was identified and deleted in recombinant strains. Then, the Weimberg pathway was optimized and overexpressed in *M. thermophila*. Growth on d-xylose was restored after construction of the Weimberg pathway in *M. thermophila*. Also, this fungus was engineered to produce BT via XOP. After that, transcript profiles were investigated. Results showed that genes related to growth were obviously upregulated in strains containing the Weimberg pathway. This work provides a way to produce xylose-derived metabolites via XOP in filamentous fungi.

## Methods

### Strains, media, and growth conditions

*M. thermophila* strains used in this study are listed in Table 1. JG207 is a promising strain to produce malic acid from cellulose, which has been engineered from WT strain by overexpressing a malate transporter-encoding gene *Aomae* and a pyruvate carboxylase *Aopyc* (more details can be seen in reference [24]). The exploration of the Weimberg pathway was performed in JG207. Considering the available resistance genes in the following construction, WT strain was selected to be engineered to produce BT. All *M*. *thermophila* strains were grown on GVMM agar plates at 35 °C for 7–10 days to obtain conidia. *E. coli* DH5α was used for construction and amplification of plasmids. *E. coli* was cultivated in Luria–Bertani (LB) medium with 100 µg/mL ampicillin for plasmid selection.

**Table 1:** Strains used in this study

Strain name	Relevant genotype	References
WT	*M. thermophila* ATCC42464 wide type	
JG207	overexpressing *Aomae*/*Aopyc*	[24]
DF1101	JG207, Δ*MYCTH_113236*	This study
DF1102	JG207, Δ*Mtxyl1*	This study
DF1135	JG207, *Mtxyl1*;Δ*Mtxyl1*	This study
DF1230	JG207, *pck*::*xylB*/*xylC*	This study
DF1232	JG207, Δ*Mtxyl1*, *pck*::*xylB*/*xylC*	This study
DF1203	JG207, Δ*Mtxyl1*, *pck*::*xylB*/*xylC*/*xylD*/*xylX*/*xylA*	This study
DF1309	JG207, Δ*Mtxyl1*, *pck*::*Trxyd1*	This study
DF1310	JG207, Δ*Mtxyl1*, *pck*::*Mtxyd1*	This study
DF1406	JG207, Δ*Mtxyl1*, overexpressing *Mtxyd1*/*xylD*/*xylX*/*xylA*	This study
DF1501	JG207, Δ*Mtxyl1*, overexpressing *Mtxyd1*/*xylD*/*BxxylX*/*KsaD*	This study
YL1134	WT, overexpressing *Mtxyd1*/*xylC*/*xylD*/*kivD*/*adh1*	This study
YL1212	YL1134, overexpressing *mdlc*	This study

For growth phenotypic profiling, starting strain JG207 and its mutants were grown on VMM with d-glucose or d-xylose containing 1.5% agar plates (90 mm diameter). All strains were inoculated with 2 µL suspension containing 500 spores and grown for 6 days at 35 °C.

For d-xylose consumption tests, two methods were implemented. One was inoculation of conidia into 50 mL VMM supplemented with 2% (w/v) d-xylose (XVMM) to a final concentration of 2.5 × 10^5^ conidia/mL in a 250-mL Erlenmeyer flask. The other was inoculation of mycelium into XVMM in a 250 mL Erlenmeyer flask. Before that, strains were pre-cultivated in GVMM for 24 h. Then the pre-cultivated medium was filtered through a Whatman glass microfiber filter on a Buchner funnel and washed thrice with VMM. Afterwards, about 2.0 g filtered mycelium (wet weight) was transferred into a shake flask with 50 mL XVMM. Dry cell weight (DCW) was used to calculate growth rate. All liquid cultures mentioned above were incubated under 45 °C at 150 rpm in a rotary shaker.

For BT production, a fermentation medium was used, containing (per liter) 40 g d-xylose, 5 g glucose, 8 g Bacto peptone, 4 g yeast extract, 0.15 g KH_2_PO_4_, 0.15 g K_2_HPO_4_, 0.12 g MgSO_4_·7H_2_O, 0.1 g CaCl_2_, 0.1 g thiamine hydrochloride, 1 mL biotin (0.1 g/L), 1 mL trace element solution (3.7 g/L (NH_4_)_6_(Mo7O_24_)·4H_2_O, 2.9 g/L ZnSO_4_·7H_2_O, 24 g/L H_3_BO_3_, 2.5 g/L CuSO_4_·5H_2_O, 15.8 g/L MnCl_2_·4H_2_O, 2 g/L Fe(NH_4_)_2_(SO_4_)_2_·6H_2_O, 5 g/L citric acid. BT fermentation was carried out in 100-mL medium in 250-mL Erlenmeyer flasks under 40 °C at 150 rpm in a rotary shaker.

### Construction of expression and deletion plasmids

All primer sequences and plasmids used in this study are listed in Additional file 9 and Additional file 10. For *MYCTH_43671* and *MYCTH_113236* deletion, vectors carrying donor DNA were constructed. 5′- and 3′-flanking regions were amplified from *M. thermophila* genomic DNA. PtrpC-*neo* was amplified from plasmid pAN52-PtrpC-*neo* [24]. pAN52 backbone containing ampicillin resistance gene (*ampR*) was amplified from plasmid pAN52-PtrpC-*neo*. 5ʹ- and 3ʹ-flanking fragments of the target genes and selectable marker cassette were assembled using the NEB Gibson assembly kit to generate plasmids donor-pAN52-43671-*neo* and donor-pAN52-113236-*neo*. Also, a donor DNA sequence without marker cassette was designed by introducing stop codon (TAA) centrally in the flank sequences named donor-43671-TAA for seamless gene deletion. For complementation of *MYCTH_43671*, a plasmid donor-pAN52-43671-*bar*-comp containing promoter of *MYCTH_43671* (predicted by Promoter-2.0 [44]) and whole gene sequence of *MYCTH_43671*. In detail, PtrpC-*bar* was amplified from pAN52-TB-Intron [45]. Fragment containing *MYCTH_43671* and its promoter was amplified from *M. thermophila* genomic DNA. Then these two fragments were ligated with fragment pAN52-5′-3′-43671 which was amplified from donor-pAN52-43671-*neo*.

Heterologous genes used in this study were codon-optimized for *M. thermophila* and synthesized from Genewiz Biotechnology (Tianjin, China). The synthesized genes were delivered on pUC-GW-Amp vectors and the optimized sequences were listed in Additional file 11. In order to integrate the upper Weimberg pathway *xylB* and *xylC* into *pck* (*MYCTH_2315623*) locus, a plasmid donor-pAN52-pck-*xylBC*-*neo* was constructed. Detailly, fragment *xylC*-Tpdc was created by overlapping PCR, digested with *Spe*I and *Eco*RV, and ligated with pAN52-PtrpC-*neo*-PgpdA [24] digested with the same restriction enzymes, resulting in plasmid pAN52-*xylC*-*neo*. Then, fragment Ppgk-*xylB* was amplified using overlapping PCR, digested with *Bam*HI and *Eco*RV, and inserted into pAN52-*xylC*-*neo* digested with the same restriction enzymes, obtaining plasmid pAN52-*xylBC*-*neo*. 5′- and 3′-flanking regions of *pck* were amplified from *M. thermophila* genomic DNA. After that, the flanking regions were assembled into pAN52-*xylBC*-*neo* digested with *Pac*I and *Nde*I to generate donor DNA sequence donor-pck-*xylBC*-*neo*.

The lower part of the Weimberg pathway containing *xylD*, *xylX* and *xylA* was designed to insert *neo* locus after *xylB*. Hence, a plasmid donor-pAN52-*xylDXA*-*bar* was assembled. The coding regions for xylD, xylX and xylA were inserted in between the promoter–terminator pairs of Ppdc and TgpdA, Peif and Tcbh, Ptef and Teif, respectively. TtrpC-PtrpC-*bar* was amplified from pAN52-TB-Intron. With the aid of the NEB Gibson assembly kit, the fragments TtrpC-PtrpC-*bar*, Ppdc-*xylD*-TgpdA, *xylA*-Teif, and 3′-flanking regions of *pck* were ligated with pAN52-TB-Intron digested with *Pac*I and *Nde*I to form plasmid pAN52-*xylDA*-bar. Then, fragments Peif-*xylX*-Tcbh and Peif were assembled into pAN52-*xylDA*-*bar* digested with *Pme*I to obtain plasmid donor-pAN52-*xylDXA*-*bar*.

To optimize XDH, another two candidate genes were employed. In detail, *Trxyd1* (TRIREDRAFT_53673) was amplified from *Trichoderma reesei* QM6a cDNA and *Mtxyd1* (*MYCTH_11247*), a homologous gene of *Trxyd1*, was cloned from cDNA of *M. thermophila*. These two sequences were, respectively, ligated with fragment donor-pAN52-*pck* which was amplified from plasmid donor-pAN52-*pck*-*xylBC*-*neo*, to form plasmid donor-pAN52-*pck*-*Trxyd1* and donor-pAN52-*pck*-*Mtxyd1* with the same promoter and terminator as *xylB*.

For overexpressing the Weimberg pathway genes, plasmids pAN52-*Mtxyd1*-*xylD*-*neo*, pAN52-*xylX*-*xylA*-*neo*, pAN52-*xylX*-*KsaD*-*neo* and pAN52-*BxxylX*-*KsaD*-*neo* were constructed. Fragment *xylD*-Tgpda was amplified from donor-pAN52-*xylDXA*-*bar*. Fragment Ptef-*Mtxyd1*-TtrpC was amplified using overlapping PCR. The two fragments were ligated with pAN52-PtrpC-*neo*-PgpdA digested with *Spe*I and *Xba*I to form plasmid pAN52-*Mtxyd1*-*xylD*-*neo*. PtrpC-*neo* was amplified from pAN52-PtrpC-*neo*-PgpdA and digested with *Hpa*I and *Not*I. Then the digested fragment was inserted into donor-pAN52-*xylDXA*-*bar* digested with the same restriction enzymes, obtaining plasmid pAN52-*xylX*-*xylA*-*neo*. Ptef-*KsaD*-Teif was amplified using overlapping PCR. pAN52-*neo* backbone was amplified from plasmid pAN52-PtrpC-*neo*. Peif-*BxxylX*-Tcbh was amplified using overlapping PCR. Then, these three fragments were assembled into plasmid pAN52-*BxxylX*-*KsaD*-*neo*.

For construction of sgRNA expression plasmids, specific sgRNA target sites in *MYCTH_43671* and *MYCTH_113236* were identified using the sgRNACas9 tool [46] with *M. thermophila* genome sequence and the target gene as inputs. Oligos with low off-target probability were selected and protospacer sequences are shown in Additional file 9. *M. thermophila* U6 promoter, RNA polymerase III U6 snRNA gene and pJET1.2/blunt backbone were directly amplified from plasmid U6-pck-sgRNA [24]. Then, the fragments were assembled by NEB Gibson assembly kit to form plasmids U6-43671-sgRNA, and U6-113236-sgRNA. The Cas9-expression PCR cassette Ptef1-*Cas9*-TtprC was amplified from plasmid p0380-*bar*-Ptef1-*Cas9*-TtprC using Ptef-cas-F/TtprC-cas-R [27].

For engineering *M. thermophila* to produce BT, three plasmids were constructed. Fragments Ppsf-*Mtxyd1*-Ttrpc, Pcwg-*xylC*-Tpdc and Pahr-*adh1*-Tcdh were created by overlapping PCR. These three fragments were ligated with pAN52-PtrpC-*neo* resulting in the first plasmid pAN52-*Mtxyd1*-*xylC*-*adh1*-*neo* using NEB Gibson assembly kit. Fragments Phsp70-*xylD*-Tpsf and Pcyc-*KivD*-Teif5A were constructed via overlapping PCR and assembled into plasmid pCSN44 digested with *Hind*III and *Bcu*I to obtain the second plasmid pCSN44-*xylD*-*KivD*-*hygr*. Fragment *mdlC* was inserted into pAN52-PtrpC-*bar*-PgpdA-TtprC digested with *Spe*I and *Eco*RV resulting in the third plasmid pAN52-*mdlC*-*bar*.

All the constructed plasmids were verified by sequencing. The restriction enzymes and T4 DNA ligase were purchased from Thermo Scientific and DNA polymerase was purchased from Vazyme Biotech Co., Ltd.

### Transformation of *Myceliophthora* protoplasts

PEG-mediated protoplast transformation was used for heterologous expression of interesting genes or gene disruption in *M. thermophila* as previously described [40]. For gene disruption by the CRISPR/Cas9 system, 10 μg Cas9-expression PCR cassette Ptef1-Cas9-TtprC, gRNA expression PCR cassette, and the corresponding donor fragment were mixed at a molar concentration ratio of 1:1:1 and added to fungal protoplasts. For gene overexpression, 10 μg PCR products or linearized vector were used. The transformants were grown for 3 days on GVMM agar plates at 35 °C with selection for *neo* resistance using geneticin (100 μg/mL), *bar* resistance using phosphinothricin (100 µg/mL) and *hph* resistance using hygromycin B (50 µg/mL). The presence of the transgenes was verified by PCR.

### In vitro enzyme activity measurements

Conidia were inoculated in 50-mL shake flasks containing 50 mL GVMM to a final concentration of 2.5 × 10^5^ conidia/mL and grown at 45 °C and 150 rpm for 24 h. Mycelia were harvested using vacuum filtration and washed three times with distilled water and immediately frozen in liquid nitrogen. The samples were stored at − 80 °C until following analysis. For protein extraction, the frozen mycelia were ground into powder with liquid nitrogen in a mortar. The powder was transferred into l mL 10 mM phosphate buffered saline pH 7.4 (PBS) with addition of 10 μg/mL phenylmethylsulphonyl fluoride (PMSF). After centrifugation for 10 min at 4 °C, 10,000 × g, clear supernatant was used for total protein quantification and enzyme assay.

Total protein in supernatant was quantified by the Bio-Rad Protein Assay Kit (Bio-Rad) with bovine serum albumin as the standard at 595 nm. Enzyme activity was assayed by following the rate of NAD^+^ or NADP^+^ reduction with measuring the optical density at 340 nm (OD340 nm, SpectraMax M5, Molecular Devices). The assays were performed in triplicate with final volume of 200 µL at 30 °C using UV-compatible 96-well plates (Corning, Germany). The standard assay mixture for all the enzymatic reactions contained 100 mM Tris–HCl pH 8.0, 2 mM MgCl_2_ and 2 mM NAD^+^ or NADP^+^ with addition of 10 µL protein extract. A concentration of 100 mM of d-xylose was used as substrate for XDH XylB, Trxyd1 and Mtxyd1 [10]. The overall combined activity of the lower part of the pathway including XylD, XylX/BxXylX and XylA/KsaD was assayed by following the formation of NADH using 10 mM d-xylonate as substrate [28].

### Metabolite analysis and mycelium dry weight determination

The concentration of d-xylose and BT was determined by HPLC (e2695; Waters, Manchester, United Kingdom) equipped with a Waters 2414 refractive index (RI) detector and an Aminex HPX-87H column (Bio-Rad) at 35 °C; 5 mM H_2_SO_4_ was used as the mobile phase with constant flow rate 0.5 mL/min. d-Xylonate was monitored with a Waters 2489 UV detector. For extracellular metabolite analysis, 1-mL sample was centrifuged at 10,000 × g for 2 min and the supernatant was stored at − 20 °C before analysis. For determination of intracellular metabolite, mycelia were harvested using vacuum filtration and washed three times with distilled water and immediately frozen in liquid nitrogen. The samples were stored at − 80 °C until following analysis. For intracellular metabolite extraction, the frozen mycelia were ground into powder in liquid nitrogen in a mortar. The powder was transferred into l mL 10 mM PBS. After centrifugation for 10 min at 10,000 × g, clear supernatant was collected. The supernatant was filtered with 0.2-μm cellulose acetate syringe filters before HPLC analysis.

GC–MS assay was also carried out for metabolite analysis. For extracellular metabolite, 5–10 μL supernatant was used. For measurement of intracellular metabolite, mycelia were collected and processed as mentioned above with some modifications. The grounded powder was transferred into 1 mL of acetonitrile–methanol–water (40:40:20, v:v:v) and centrifuged for 10 min at 10,000 × g under 4 °C. An equal amount of each sample was taken for determination which used total protein concentration to normalize the weight of mycelia. Samples were dried using vacuum concentrator at 4 °C. The dried samples were dissolved in 50 μL of pyridine containing 40 mg/mL methoxyamine hydrochloride, and incubated for 90 min at 30 °C. Then, 50 μL of N-methyl-N-trimethylsilyltrifluoroacetamide (MSTFA) reagent (containing 1% trimethylchlorosilane (TMCS), v/v) was added to the sample aliquots incubated for 60 min at 37 °C. After centrifugation for 5 min at 10,000 × g, clear supernatant was assayed by GC–MS as described previously [47].

For determination of biomass dry weight, 1 mL sample was taken and filtered using vacuum filtration. Then the filtered mycelium was washed three times with distilled water and dried at 80 °C until constant weight. All samples were analyzed in triplicate.

### Transcriptome analysis

Strains were pre-grown in 50-mL shake flasks containing 50 mL GVMM at 45 °C and 150 rpm for 24 h. Then mycelia were filtered using a Whatman glass microfiber filter on a Buchner funnel and washed three times with VMM. The filtered mycelia were transferred into shake flasks containing 50 ml VMM with 1% (w/v) d-xylose and cultured for 4 h. After that, mycelia for RNA isolation were harvested using vacuum filtration. The collected mycelia were washed thrice with distilled water and immediately frozen in liquid nitrogen. The samples were stored at − 80 °C until RNA extraction. Total RNA was extracted using TRIzol reagent method described previously [48]. Total RNA sample from each strain were purified with a Qiagen RNeasy Mini kit. RNA sequencing (RNA‐seq) was performed using DNBSEQ platform at BGI (Shenzhen, China).

Sequenced reads were trimmed and filtered with SOAPnuke [49]. Clean reads were mapped against *M. thermophila* ATCC 42464 genome v2.0 [50] (http://genome.jgi.doe.gov/) with < 2-base mismatching, using TopHat (v2.0.12) [51]. Raw counts of reads that uniquely mapped to only one gene were calculated for each gene by HTSeq (v0.13.5) [52] and used for normalizing transcript abundance (Reads Per Kilobase per Million mapped reads, RPKM, Additional file 12). Differential gene expression was analyzed using DEseq2 package (v1.30.1) [53]. The RNA-seq raw data were deposited in the Gene Expression Omnibus (accession number: GSE214002) at the National Center for Biotechnology Information (NCBI).

### Determining copy numbers by RT-qPCR

To determine copy number of the inserted genes in genome of *M. thermophila*, fungal genomic DNA was extracted as described previously [54]. The diluted DNA (20–50 ng/μL) was used as template for RT-qPCR. RT-qPCR was performed using the SYBR Green Realtime PCR Master Mix (TOYOBO, Osaka, Japan) with *actin* gene (*MYCTH_2314852*) as an internal control [27] using CFX96 Real-Time PCR Detection System (Bio-Rad, Hercules, CA, USA). The primers used for the genes are listed in Additional file 9.

## Supplementary Information

**Additional file 1. Fig. S1**: d-Xylose consumption of strains DF1102, DF1231, and DF1203 inoculated with 2 g (wet weight) mycelium (**a**). HPLC peak (UV detector, 210 nm) of extracellular metabolites at day 6 (**b**) and intracellular metabolites at day 4 (**c**).

**Additional file 2. Fig. S2**: Growth phenotype of JG207 and DF1230 on VMM agar plates containing d-glucose, d-xylose, and mixture of 20 g/L d-xylose and 5 g/L d-glucose.

**Additional file 3. Fig. S3**: **a** HPLC peak (UV detector, 210 nm) of extracellular metabolites at day 6. **b** GC–MS analysis of extracellular metabolites at day 9. **c** MS spectra of d-xylonate at 17.505 min.

**Additional file 4. Fig. S4**: **a** Copy number of the Weimberg pathway genes. **b** Transcript level of Mtxyd1 in *M. thermophila* mutants.

**Additional file 5. Fig. S5**: GC–MS peak area intensity of intracellular metabolites at 6 h inoculated with 2 g (wet weight) mycelium. **a** Peak area intensity of d-xylonate at *m*/*z* = 277.1446. **b** Peak area intensity of α-ketoglutarate at *m*/*z* = 198.0581.

**Additional file 6. Fig. S6**: Specific activity of the lower part of the Weimberg pathway including XylD, XylX/BxXylX and XylA/KsaD in different recombinant *M. thermophila* strains.

**Additional file 7**: The flux distribution in oxo-reduction and the Weimberg pathway predicted by GEM of *M. thermophila* iDL1450.

**Additional file 8. Fig. S7**: Growth analysis of JG207 (**a**) and DF1501 (**b**) in flask inoculated with 2 g (wet weight) mycelium.

**Additional file 9**: Primers used for the genetic manipulation in *M. thermophila*.

**Additional file 10**: Plasmids used in present study.

**Additional file 11**: The sequences of heterologous genes used in this study.

**Additional file 12**: The profiles of transcriptional data.

## Data Availability

All data generated or analyzed during this study are included in this published article and its additional files.

## Abbreviations

PPP: Pentose phosphate pathway
TCA: Tricarboxylic acid cycle
XOP: Xylose oxidative pathway
XDH: D-Xylose dehydrogenase
XL: D-Xylonolactonase
XD: D-Xylonate dehydratase
KdxD: 2-Keto-3-deoxy-d-xylonate dehydratase
BT: 1,2,4‐Butanetriol
VMM: 1 × Vogel’s minimal medium
XVMM: VMM supplemented with 2% (w/v) d-xylose
GVMM: VMM supplemented with 2% (w/v) glucose
LB: Luria–Bertani
PBS: Phosphate buffered saline
HPLC: High-performance liquid chromatography
GC–MS: Gas chromatography–mass spectrometry
CAZymes: Carbohydrate-active enzymes
LPMO: Lytic polysaccharide monooxygenases
DCW: Dry cell weight
GEM: Genome-scale metabolic model
FBA: Flux balance analysis
