# Supplementary figures and images for: The Weimberg pathway: an alternative for Myceliophthora thermophila to utilize d-xylose
Source: Biotechnol Biofuels Bioprod. 2023 Jan 23;16:13. doi: 10.1186/s13068-023-02266-7 (PMC9869559; doi:10.1186/s13068-023-02266-7)

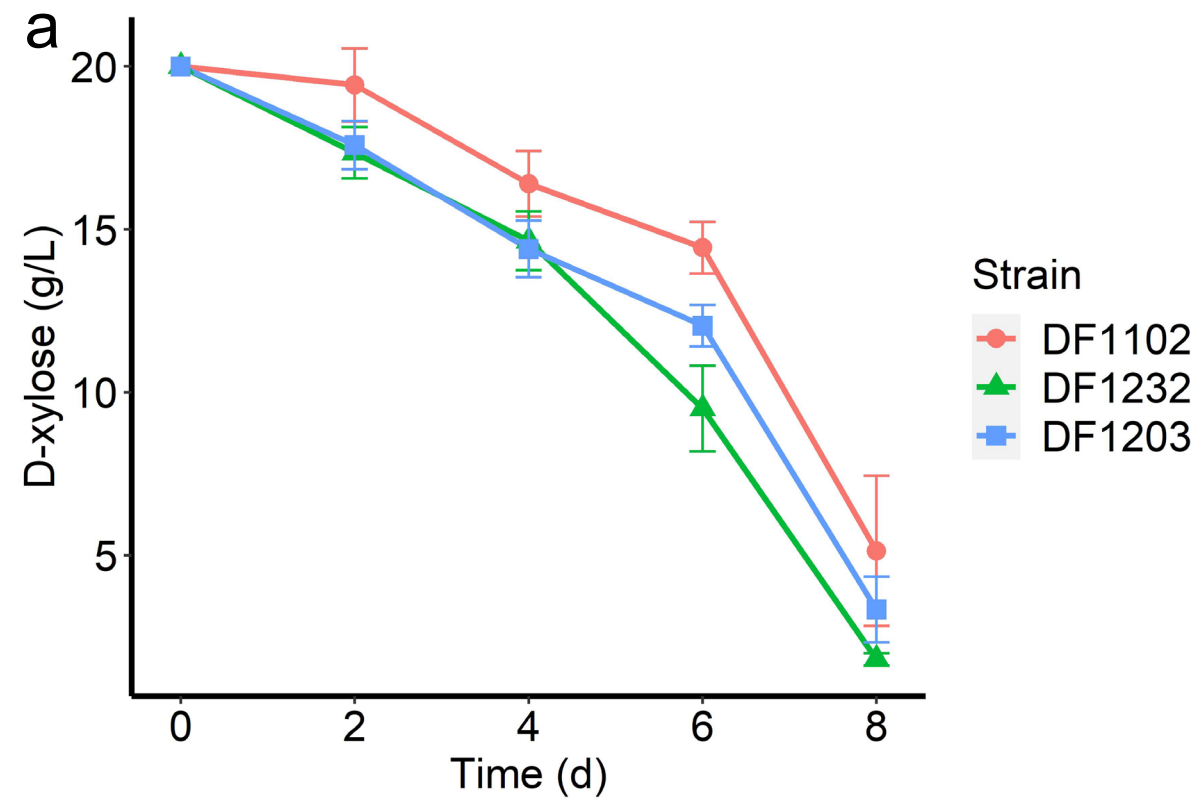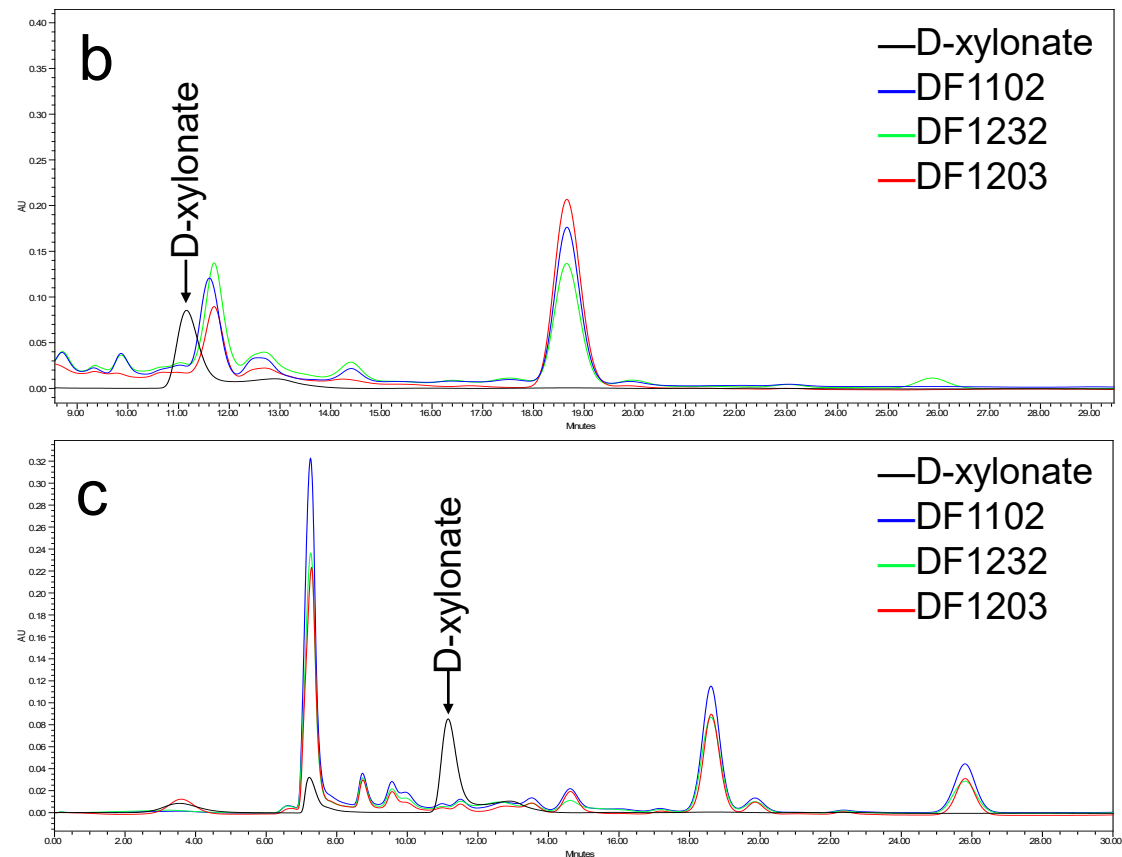

Supplement: Supplementary file 1 — Additional file 1. Fig. S1: d-Xylose consumption of strains DF1102, DF1231, and DF1203 inoculated with 2 g (wet weight) mycelium (a). HPLC peak (UV detector, 210 nm) of extracellular metabolites at day 6 (b) and intracellular metabolites at day 4 (c). [file 13068_2023_2266_MOESM1_ESM.pdf]

JG207

DF1230

D-glucose

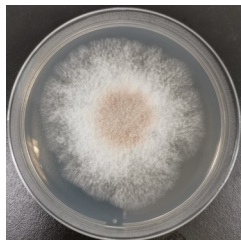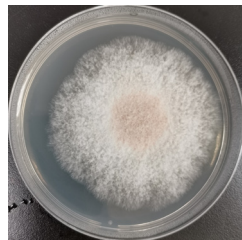

D-xylose

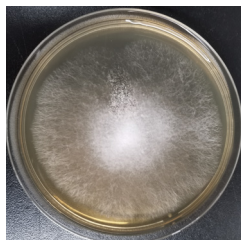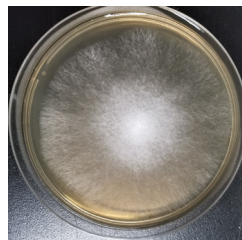

D-xylose +  
D-glucose

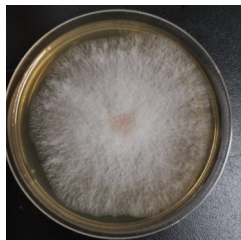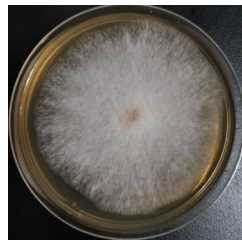

Supplement: Supplementary file 2 — Additional file 2. Fig. S2: Growth phenotype of JG207 and DF1230 on VMM agar plates containing d-glucose, d-xylose, and mixture of 20 g/L d-xylose and 5 g/L d-glucose. [file 13068_2023_2266_MOESM2_ESM.pdf]

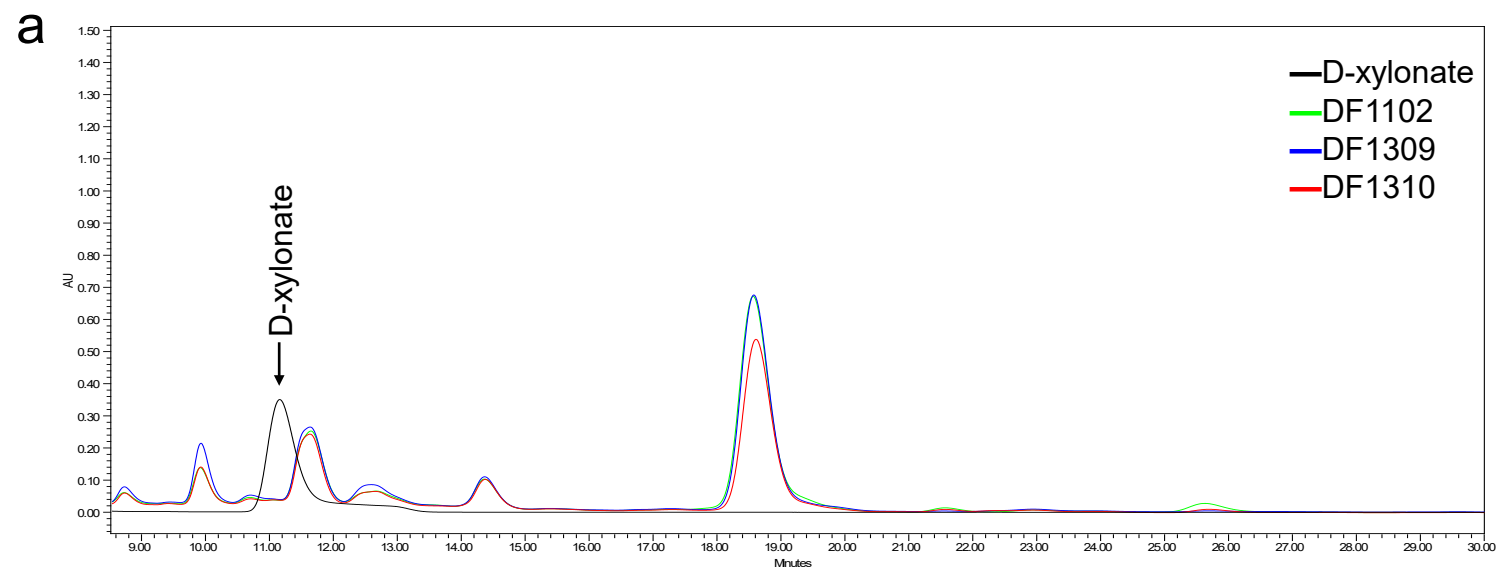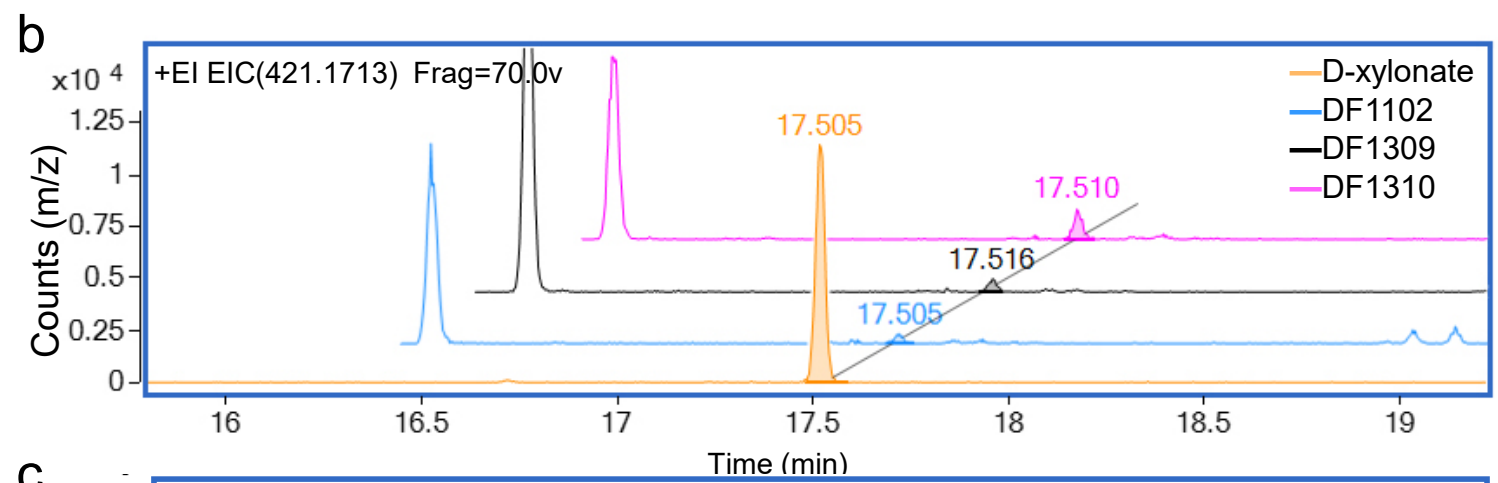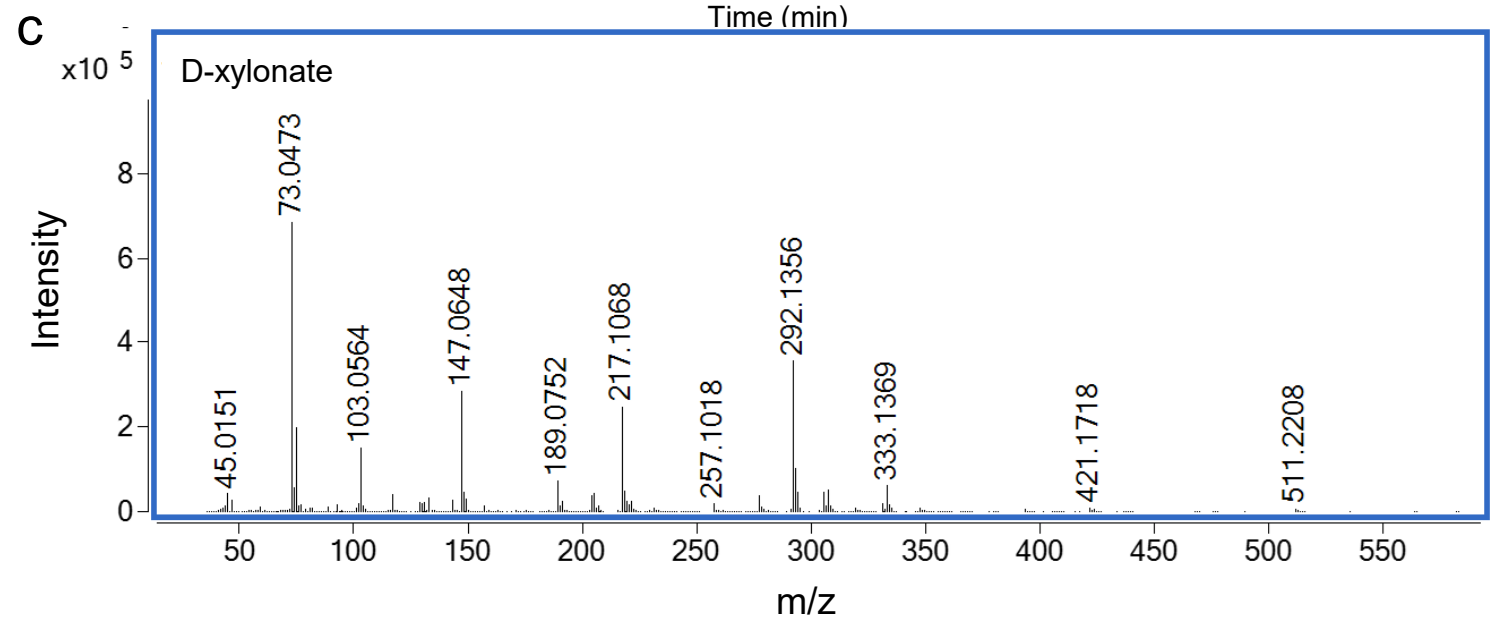

Supplement: Supplementary file 3 — Additional file 3. Fig. S3: a HPLC peak (UV detector, 210 nm) of extracellular metabolites at day 6. b GC–MS analysis of extracellular metabolites at day 9. c MS spectra of d-xylonate at 17.505 min. [file 13068_2023_2266_MOESM3_ESM.pdf]

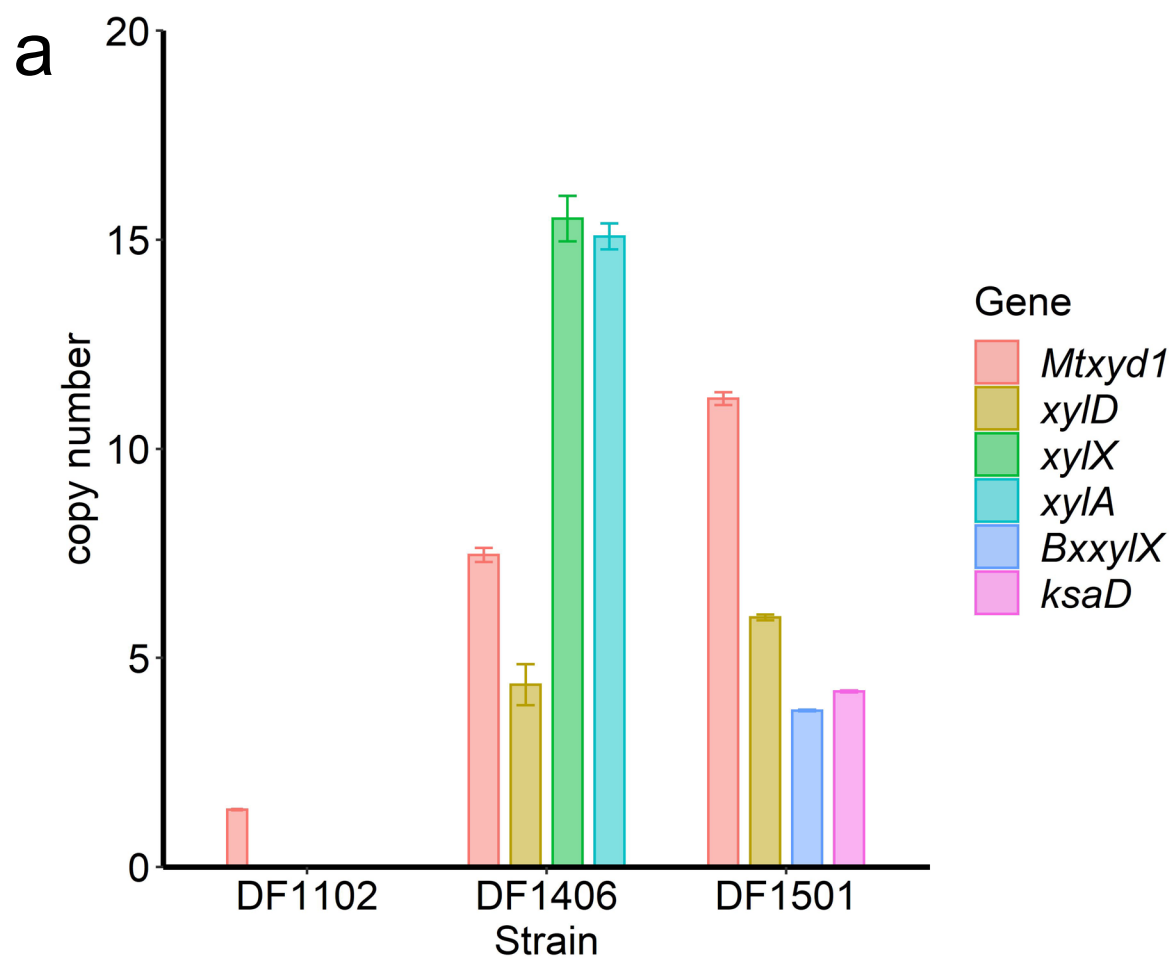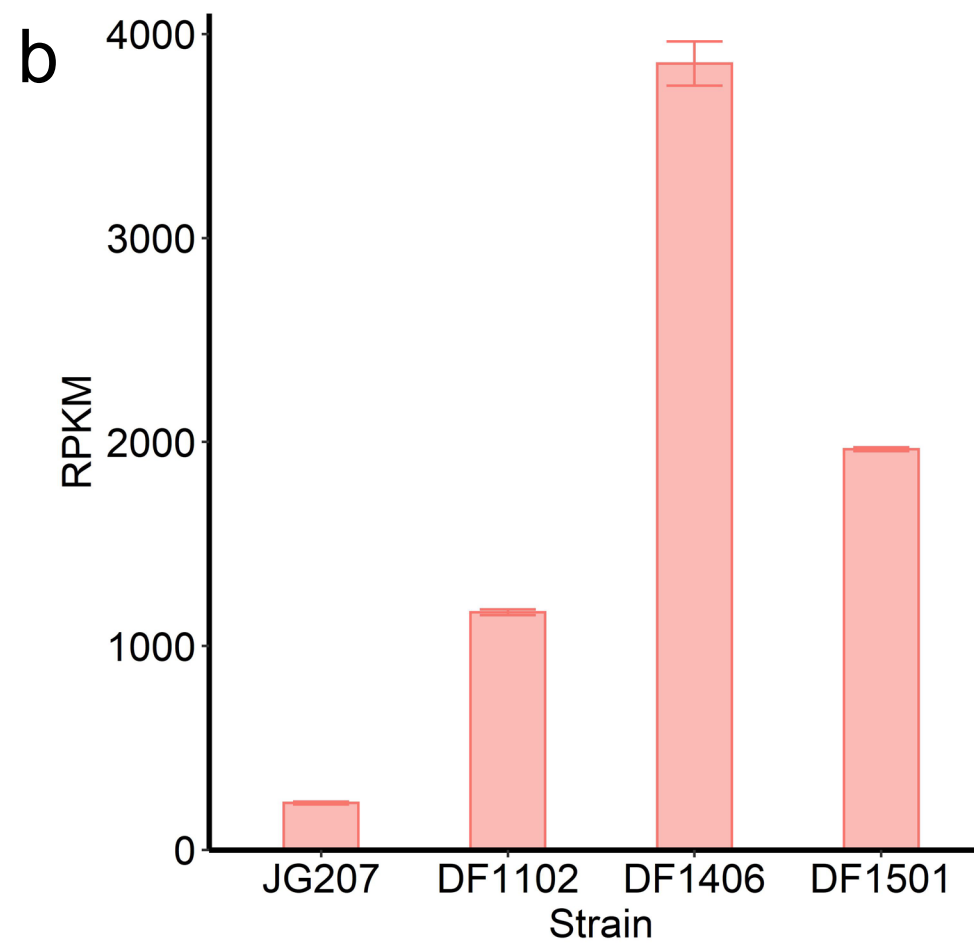

Supplement: Supplementary file 4 — Additional file 4. Fig. S4: a Copy number of the Weimberg pathway genes. b Transcript level of Mtxyd1 in M. thermophila mutants. [file 13068_2023_2266_MOESM4_ESM.pdf]

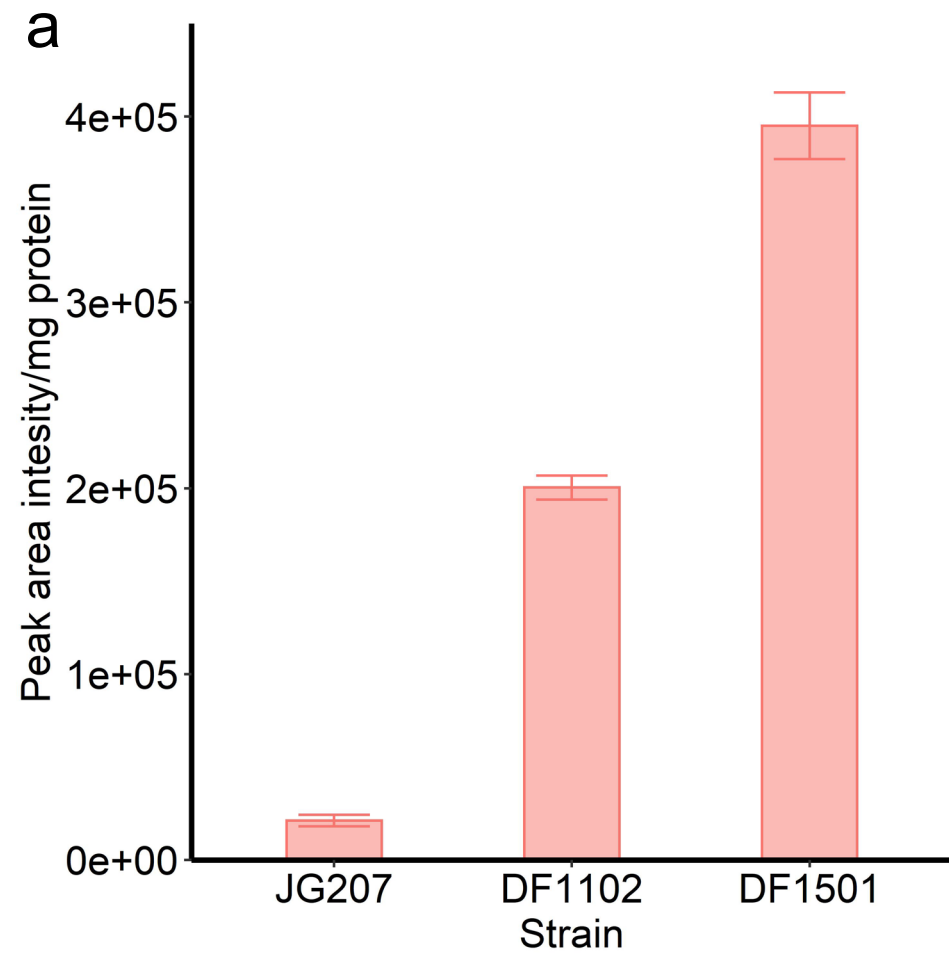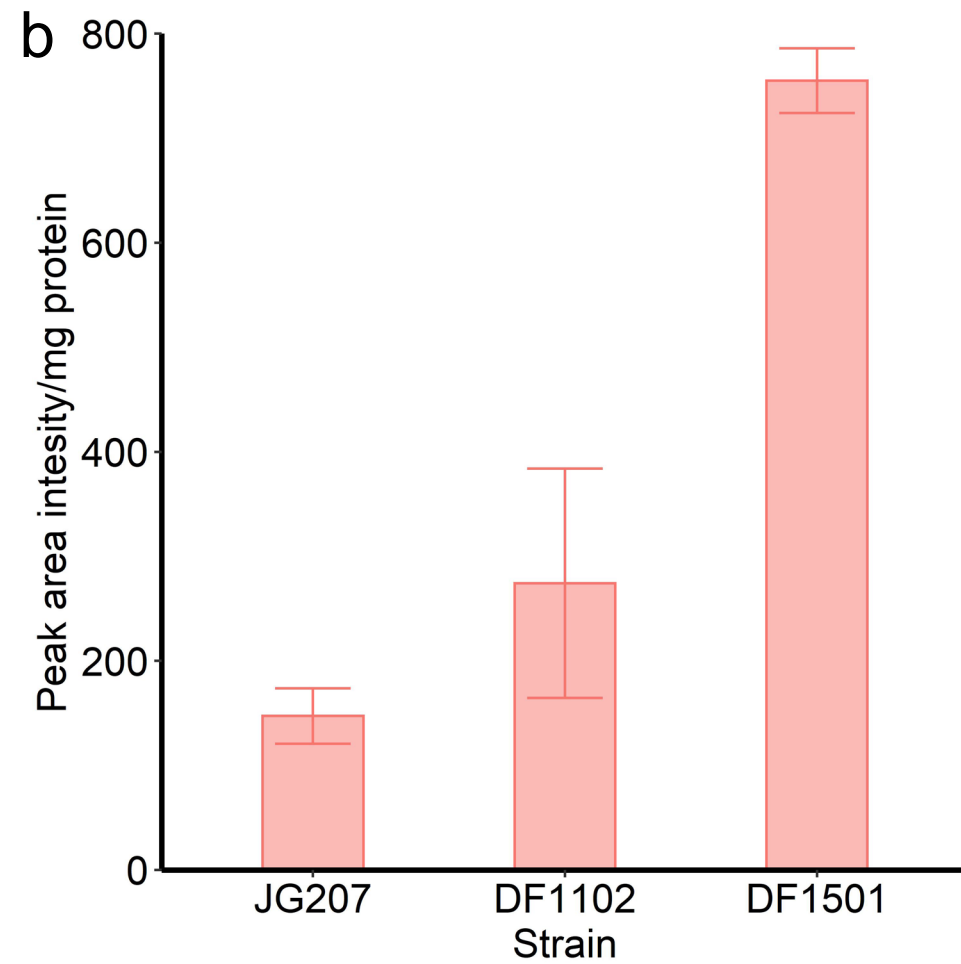

Supplement: Supplementary file 5 — Additional file 5. Fig. S5: GC–MS peak area intensity of intracellular metabolites at 6 h inoculated with 2 g (wet weight) mycelium. a Peak area intensity of d-xylonate at m/z = 277.1446. b Peak area intensity of α-ketoglutarate at m/z = 198.0581. [file 13068_2023_2266_MOESM5_ESM.pdf]

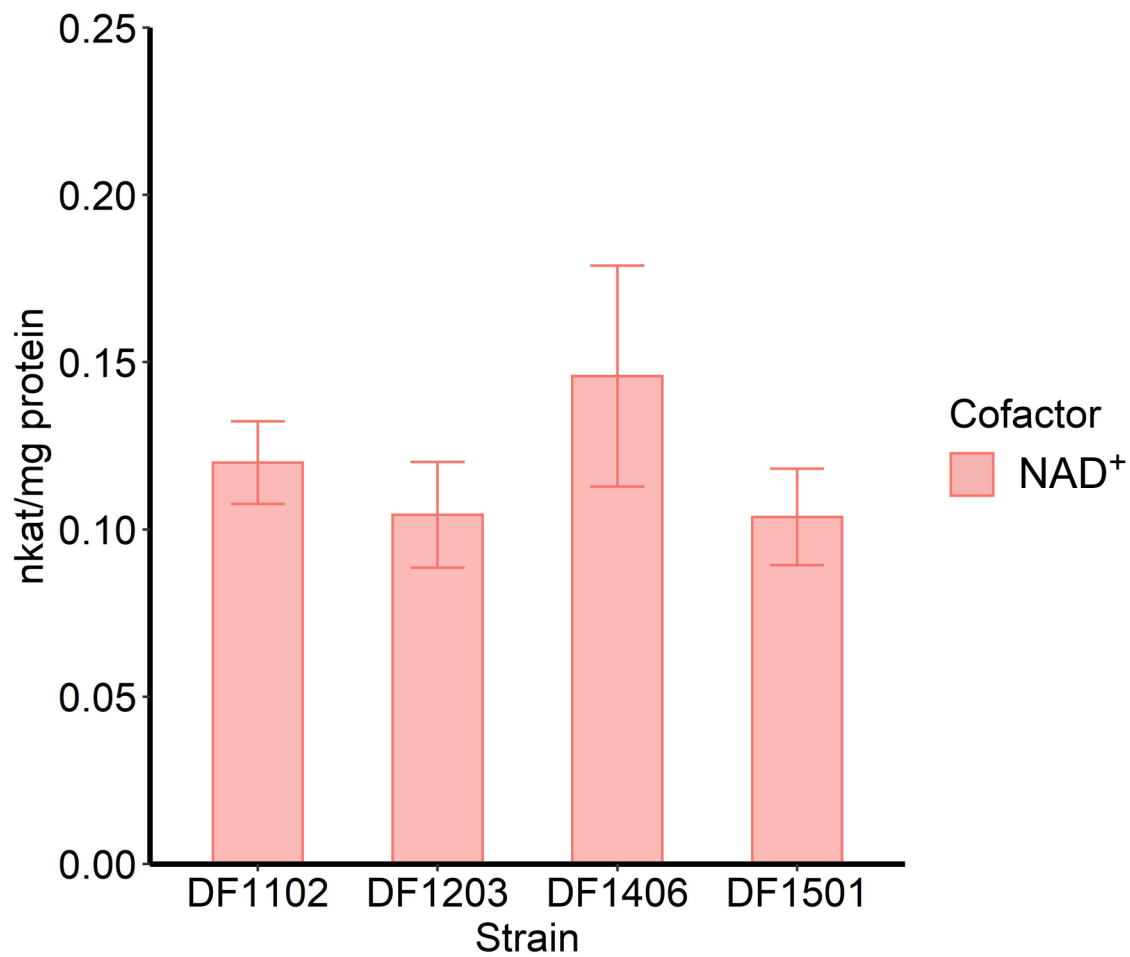

Supplement: Supplementary file 6 — Additional file 6. Fig. S6: Specific activity of the lower part of the Weimberg pathway including XylD, XylX/BxXylX and XylA/KsaD in different recombinant M. thermophila strains. [file 13068_2023_2266_MOESM6_ESM.pdf]

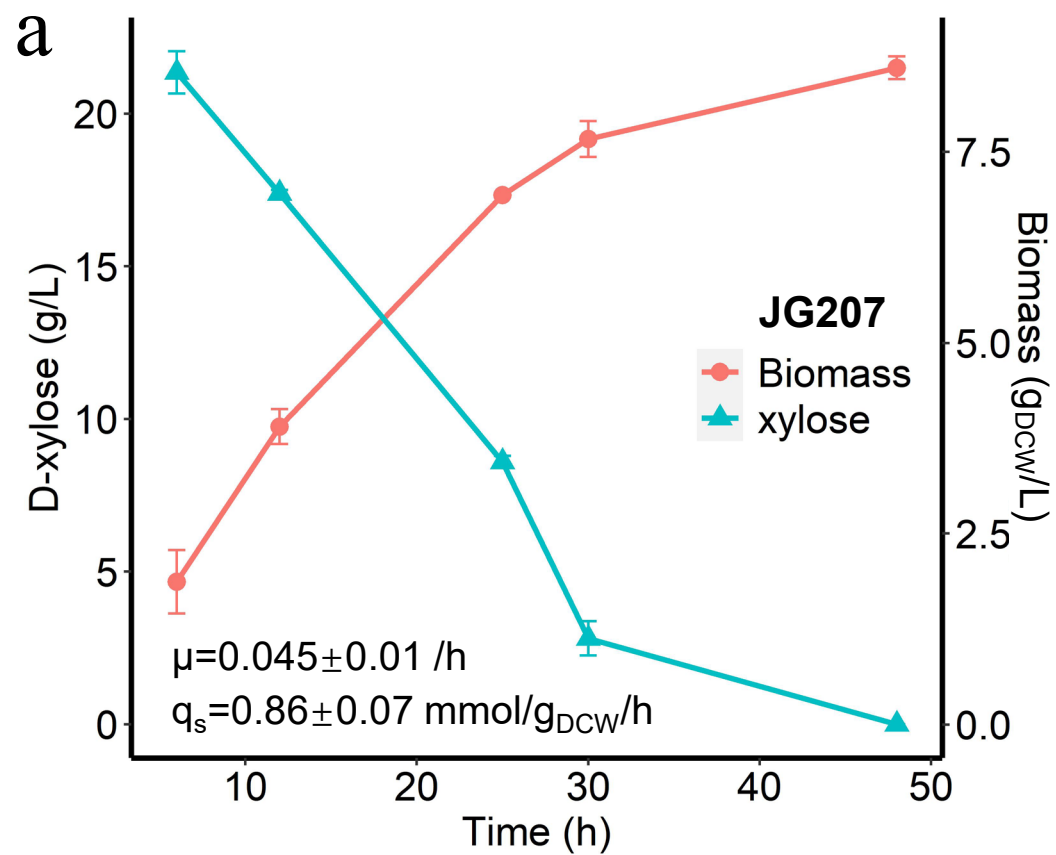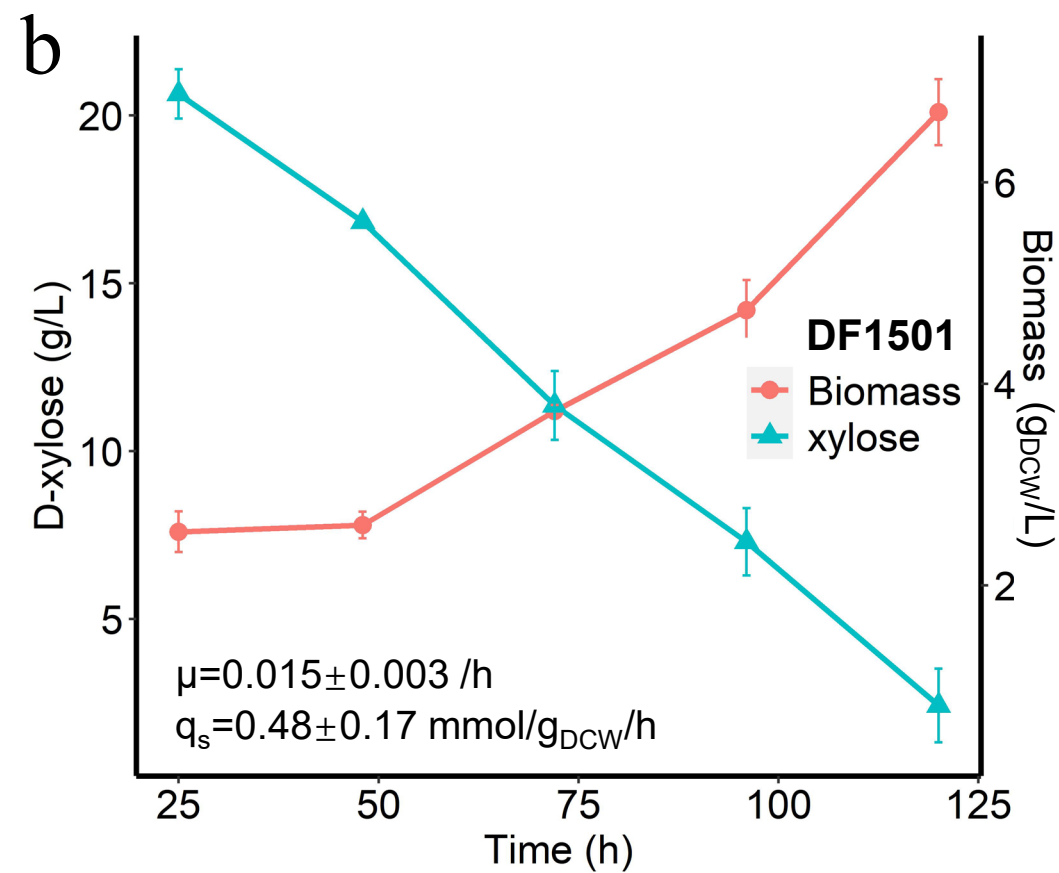

Supplement: Supplementary file 8 — Additional file 8. Fig. S7: Growth analysis of JG207 (a) and DF1501 (b) in flask inoculated with 2 g (wet weight) mycelium. [file 13068_2023_2266_MOESM8_ESM.pdf]
