# Supplementary material for: The Weimberg pathway: an alternative for Myceliophthora thermophila to utilize d-xylose
Source: Biotechnol Biofuels Bioprod. 2023 Jan 23;16:13. doi: 10.1186/s13068-023-02266-7 (PMC9869559; doi:10.1186/s13068-023-02266-7)
Supplement: Supplementary file 11 — Additional file 11: The sequences of heterologous genes used in this study. [file 13068_2023_2266_MOESM11_ESM.docx]

Additional file 11 The sequences of heterologous genes used in this study.

>WP_010918706.1 SDR family oxidoreductase [Caulobacter vibrioides]. The optimized sequence of *xylB* for Weimberg pathway construction. ATGTCTTCTGCAATCTACCCATCTCTAAAGGGTAAGAGGGTTGTGATCACCGGAGGAGGATCTGGAATCGGAGCAGGACTAACCGCAGGATTTGCACGGCAAGGAGCAGAAGTGATCTTCCTAGACATCGCAGATGAAGATTCTCGGGCACTAGAAGCAGAACTAGCAGGATCTCCAATCCCACCAGTGTACAAACGGTGCGATCTAATGAACCTAGAAGCAATCAAAGCAGTGTTTGCAGAAATCGGAGATGTGGATGTGCTAGTGAACAACGCAGGAAACGATGATCGGCATAAACTAGCAGATGTGACCGGAGCATACTGGGATGAACGGATCAACGTGAACCTACGGCACATGCTATTCTGTACACAAGCAGTGGCACCAGGAATGAAGAAGCGAGGCGGAGGAGCAGTGATCAACTTTGGATCTATCTCTTGGCATCTAGGACTAGAGGATCTAGTGCTATACGAAACCGCAAAGGCCGGCATCGAAGGAATGACCCGGGCACTAGCACGGGAACTAGGACCAGATGACATCCGGGTGACCTGCGTGGTGCCAGGAAACGTGAAGACTAAGCGGCAAGAGAAGTGGTATACTCCCGAGGGAGAAGCACAAATCGTGGCAGCACAATGCCTAAAGGGTCGAATCGTGCCAGAGAATGTCGCAGCACTAGTGCTATTTCTAGCATCTGATGATGCATCTCTATGCACCGGACATGAATACTGGATAGATGCAGGATGGCGGTGA

>WP_010918705.1 SMP-30/gluconolactonase/LRE family protein [Caulobacter vibrioides]. The optimized sequence of *xylC* for Weimberg pathway construction.

ATGACCGCACAAGTGACCTGCGTGTGGGATCTAAAGGCTACGCTAGGAGAAGGACCAATCTGGCATGGAGATACCCTATGGTTTGTGGACATCAAACAACGGAAGATACACAACTACCACCCGGCTACTGGCGAGCGTTTCTCGTTTGACGCGCCAGATCAAGTGACCTTTCTAGCACCAATCGTGGGAGCGACGGGCTTTGTAGTCGGACTAAAGACAGGAATCCATCGGTTTCACCCTGCTACGGGATTCTCTCTACTACTAGAAGTGGAAGATGCAGCACTAAACAACCGGCCAAACGATGCAACCGTGGATGCACAAGGACGGCTATGGTTTGGAACCATGCATGATGGAGAAGAGAATAATTCTGGATCTCTATACCGGATGGATCTAACCGGAGTGGCACGGATGGATCGGGACATCTGCATCACCAACGGACCATGCGTGTCTCCAGATGGAAAGACTTTCTACCATACCGATACCCTAGAGAAGACGATTTACGCATTTGATCTAGCAGAAGATGGACTACTATCTAACAAACGGGTGTTTGTGCAATTTGCACTAGGAGATGATGTGTACCCAGATGGATCTGTGGTGGATTCTGAAGGATACCTATGGACCGCACTATGGGGGGGATTCGGAGCAGTGCGGTTCTCGCCTCAAGGAGATGCAGTGACCCGGATCGAACTACCAGCACCAAACGTGACCAAACCATGCTTTGGAGGACCTGATCTAAAGACATTATACTTTACCACCGCACGGAAAGGACTATCTGATGAAACCCTAGCACAATACCCACTAGCAGGAGGAGTGTTTGCAGTGCCAGTGGATGTGGCAGGACAACCACAACATGAAGTGCGGCTAGTGTGA

>ACL94327.1 xylonate dehydratase *xylD* [Caulobacter vibrioides NA1000]. The optimized sequence of *xylD* for Weimberg pathway construction.

ATGTCTAACCGGACTCCTCGCCGGTTTCGGTCTCGGGATTGGTTTGATAACCCAGATCATATAGATATGACCGCACTATACCTAGAACGGTTTATGAACTACGGAATCACTCCTGAGGAACTACGGTCTGGAAAGCCGATTATCGGAATCGCACAAACCGGATCTGACATCTCTCCATGCAACCGCATCCATCTTGATCTAGTGCAACGGGTGCGGGATGGAATCCGGGATGCAGGAGGAATCCCAATGGAATTTCCAGTGCATCCAATCTTTGAGAATTGTCGGCGGCCAACCGCAGCACTAGATCGGAACCTATCTTACCTAGGACTAGTGGAAACCCTACATGGATACCCAATAGATGCAGTGGTGCTAACCACCGGATGCGATAAGACAACGCCTGCCGGAATCATGGCAGCAACCACCGTGAACATCCCAGCAATCGTGCTATCTGGAGGACCAATGCTAGATGGATGGCATGAGAATGAGCTAGTGGGATCTGGAACCGTGATCTGGCGGTCTCGGCGGAAACTAGCAGCAGGAGAAATCACCGAAGAAGAATTTATAGATCGGGCAGCATCTTCTGCACCATCTGCAGGACATTGCAACACAATGGGAACCGCATCTACCATGAACGCAGTGGCAGAAGCACTAGGACTATCTCTAACCGGATGCGCAGCAATCCCAGCACCATACCGGGAACGTGGGCAGATGGCATACAAGACGGGTCAACGGATCGTGGATCTAGCATACGATGATGTGAAACCACTAGACATCCTAACCAAACAAGCATTTGAGAATGCGATCGCACTAGTGGCAGCAGCAGGAGGATCTACCAACGCACAACCACATATCGTGGCAATGGCACGGCATGCAGGAGTGGAAATCACCGCAGATGATTGGCGGGCAGCATACGACATCCCACTAATCGTCAACATGCAACCCGCAGGGAAGTATCTCGGAGAACGGTTTCATCGGGCAGGAGGAGCACCAGCAGTGCTATGGGAACTACTACAACAAGGACGGCTACATGGAGATGTGCTAACCGTGACCGGAAAGACTATGTCTGAGAATCTCCAAGGACGGGAAACCTCTGATCGGGAAGTGATCTTTCCATACCATGAACCACTAGCAGAGAAGGCCGGGTTTCTAGTGCTAAAGGGTAATCTATTTGATTTCGCCATTATGAAATCTTCTGTGATCGGAGAAGAATTTCGGAAACGCTACCTATCTCAACCAGGACAAGAAGGAGTGTTTGAAGCACGGGCAATCGTGTTTGATGGATCTGATGATTACCATAAACGGATCAACGATCCAGCACTAGAAATAGATGAACGGTGCATCCTAGTGATCAGAGGCGCAGGTCCAATCGGATGGCCAGGATCTGCAGAAGTGGTAAACATGCAACCACCGGATCATCTACTAAAGAAGGGCATAATGTCTCTACCAACCCTAGGAGATGGACGGCAATCTGGAACCGCAGATTCTCCATCTATCCTAAACGCATCTCCAGAATCTGCAATCGGAGGAGGACTATCTTGGCTACGGACCGGAGATACCATCCGGATTGATCTAAACACCGGACGGTGCGATGCACTAGTGGATGAAGCAACCATCGCAGCACGGAAACAAGATGGAATCCCAGCAGTGCCAGCAACCATGACACCGTGGCAAGAAATCTACCGGGCACATGCATCTCAACTAGATACCGGAGGAGTGCTAGAATTTGCAGTGAAATACCAGGATCTAGCAGCAAAACTACCTAGGCACAACCATTGA

>ACL94331.1 fumarylacetoacetate hydrolase family protein [Caulobacter vibrioides NA1000]. The optimized sequence of *xylX* for Weimberg pathway construction.

ATGGGAGTGTCTGAATTTCTACCAGAAGATTGGAAAGCAGCAACCCTACTAGGACGGATAGATTTCGGTGAAGGGCCTACACCAGTACTAGTGCGAGGAGGACGTGTGGAAGATGTTTCCAAGATAGCACCTACCGTGGCTGATCTAATGAACGCATTTCAACCAGGAGCAGTGATCCCACGCGGCGAGGATAAAGGACCACTAGAAGCACTAGACATCCGGCCAGTGTGGGAAGATCCAGATGGAGCAGCACCAGTGAAACTACTAGCACCAGTGGATCTACAATGCCTAAAGGCCGCGGGAGTGACCTTTGCAGTGTCTACCCTAGAACGGGTGATCGAAGAACGGGCACGTGGCGACGCAGGAGAAGCACTAAAGATTCGAACCCTACTAGCAGAACGGATGGGAGGTGATCTAAAATCAGTGGAGCCCGGATCTCAAGGAGCACAACGGCTAAAGGACGCGCTAATCGCAGATGGACTATGGTCTCAATACCTAGAAGTGGCGATAGGGCCCGACGCCGAAATCTTTACCAAGGGCCCTACTCTGAGTTCTATGGGATGGGGGGACCAAGTGGGAGTGCGGTACGATTCTCATTGGAACAACCCAGAACCAGAAGTGGTGCTACTATGCGATGGATCTGGACTAATCAGAGGCGCGGCGCTAGGAAACGATGTGAACCTACGGGATTTCGAGGGCCGGTCTGCACTACTACTATCTAAAGCAAAGGACAATAACGCATCTTGCGCCATAGGCCCATTCTTCCGTCTGTTTGATGAAACCTTTGGACTAGATGATGTGCGGTCTGCAGAAGTGGAACTAAAGATTACTGGACGGGATAACTTTGTGCTAGATGGAAAGTCCAATATGTCTCTAATCTCTCGCGATCCAGCAGTGCTAGCAGGACAAGCATACGGAAAGCAGCACCAATACCCAGATGGATTTGCACTATTTCTAGGAACCATGTTTGCACCAATCCAAGATCGGGATACGCCTGGCCAAGGATTTACCCATAAAGTGGGAGATCGGGTGCGGGTGTCTACACCCAAACTCGGCGTGCTAGAGAATGAGGTGACCACCTGCGATAAAGCAAAGCCTTGGACCTTTGGAATCTCTGCACTAATCCGGAACCTAGCAGGACGTGGTTTGCTATGA

>ACL94330.1 alpha-ketoglutaric semialdehyde dehydrogenase [Caulobacter vibrioides NA1000] The optimized sequence of xylA for Weimberg pathway construction.

ATGACCGATACCCTACGGCATTACATCGGAGGAGAACGGGTGGCAGCAGATGCACCAGCAGAATCTCTAAACCCATCTAACACCAACGATGTGGTGGCAAAGGTCCCGATGGGAGGACAAGCAGAAGTTGACGCTGCCGTCGACGCAGCTAGGAAGGCTTTCCCTGCCTGGGCAGATGCATCTCCAGAAGTGCGGTCTGATCTACTAGATAAAGTGGGATCTACCATCATCGCACGGTCTGCAGACATCGGACGGCTACTAGCACGGGAAGAAGGAAAGACTCTCGCAGAAGGAATCGGAGAAACCGTGCGGGCAGGACGGATCTTTAAATACTTTGCAGGAGAAGCACTACGGCGGCATGGACAGAATCTTGAATCTACCCGGCCAGGAGTGGAAATCCAAACCTACCGGCAAGCAGTGGGAGTGTACGGACTAATCACGCCGTGGAACTTTCCAATCGCAATCCCAGCATGGAAAGCAGCACCAGCACTAGCATTTGGAAACACCGTGGTGATCAAACCAGCAGGACCAACACCGGCGACCGCAAACGTGCTAGCAGACATCATGGCAGAATGCGGAGCACCAGCAGGAGTGTTTAACATGCTATTTGGACGCGGTTCGATGGGTGACGCTTTGATAAAGCATAAAGATGTGGATGGAGTGTCTTTCACGGGTTCTCAAGGAGTGGGAGCACAAGTGGCAGCAGCAGCAGTGGCACGGCAAGCACGGGTGCAACTAGAAATGGGAGGAAAGAATCCTCTAATCGTGCTAGATGATGCTGACCTAGAACGGGCAGTGGCAATCGCACTAGATGGATCTTTCTTCGCGACAGGACAACGGTGCACCGCATCTTCTCGGCTAATCGTGCAAGATGGAATCCATGATAAATTTGTGGCACTACTAGCAGAGAAGGTTGCCGCACTACGGGTAGGCGACGCGCTGGACCCGAACACCCAAATCGGACCAGCAGTGTCTGAAGATCAAATGGAAACCTCTTACCGGTACATAGACATCGCAGCATCTGAAGGAGGACGGGTGGTGACCGGAGGAGATCGGATCAAACTAGATAACCCAGGATGGTACGTGCGGCCAACCCTAATCGCAGATACCCAAGCAGGAATGCGGATCAACAACGAAGAAGTGTTTGGACCAGTGGCATCTACCATCCGGGTGAAATCTTACGAAGAAGCACTAGAAATCGCAAACGGAGTGGAATTTGGACTATCTGCAGGAATCGCAACCACCTCTCTAAAGCACGCGAGGCACTTTCAAAGATATGCACGGGCAGGAATGACAATGGTGAACCTAGCAACCGCAGGAGTGGATTACCATGTGCCATTTGGAGGAACCAAATCTTCTTCTTACGGAGCACGGGAACAAGGATTTGCGGCGGTAGAGTTCTTTACGCAGACCAAGACTTCATACTCTTGGTCTTGA

>DR64_8450 *Burkholderia xenovorans*. The optimized sequence of *BxxylX* for Weimberg pathway construction.

ATGTCTGCCACTTCCCCAAGCTCCTTCCTCCCTGATGACCTGGGCCAAGCCCTTTTAGTGGGTAGGGTCTGGAGGAAGACAGATCAGCATGAGGGTCCCTCTGTGGTGGTGGTGAGGGGTGGTGAGGTCTTTGACATCACTGCCACAGTCCCTACCACTGCTGACCTTTTTGATAGGGAGGATGCAGCCCCCTTGGCAAGGACTGCTCCTGGTGTCTCCCTAGGTGCTGTAGCTCAGCTCATAGCTTCCAATTTACCTGGTGCTGCTGCCCCTGCCTTGAGACTACTGGCCCCCTGTGATGTCCAAGCCATCAAGGCCTGTGGTGTCACCTTTGCTGTCTCCCTCATTGAGAGGGTCATTGAGGAGCAAGCTGCTGGTGACCCTGCCAAGGCCAAAGAGGTGAGGGACACCATTGCCTCCATGATTGGCACTGACCTCTCCAAGATTGAGCCTGGCTCTGATGCTGCCATGAAACTCAAAGCTGAGCTGGAGAGGAGGGGTGCATGGTCTCAGTATATGGAGGTGGGCATTGGCCCTGATGCTGAGGTCTTCTCCAAGTCTCAGCCAATGTCTGCTGTGGGCTTTGGAGCTGATGTGGGCCTGCTGGCTGCCTCCACCTGGAACAACCCTGAGCCTGAGATTGTCCTGGCTGTCAACTCTAGGGGTGAGATTGTGGGTGCCACCCTGGGCAATGATGTCAACCTGAGGGACATTGAGGGTAGGTCTGCCCTCCTCCTGGGCAAGTGCAAGGACAACAATGGCTCCTGTGCCATTGGCCCCTTTGTGAGGCTCTTTGATGAGTCCTTCTCCCTGGACTCAGTGAGGACTGCCTCTGTGGCCCTGAGGGTGGAGGGTGCTGATGATGGCTTTGTCCTGGAGGGTGTCTCCCACATGTCTGAGATTTCTAGGGACCCTGCCAACCTGGTGGCTCAGACCTGGGGTAGGCACCACCAATATCCTGATGGCTTCATGCTCTTCCTGGGCACCATGTTCTCCCCAATCAAGGATAGGGATGCCCCTGGTGCTGGCTTCACCCACCACCTGGGTGACCTGGTCACCATCTCCACCCCTCAGCTGGGTGCCCTCACCAACACTGTGAGGTTGTCTACTGAAATTGAGCCCTGGACCTTTGGTGTGAGGGCCCTCTATAGGAACCTGGCTGCTAGGGGCCTCCTGGCCTCTGCCTAA

>Cg0535 CAF19168.1 ketoglutarate semialdehyde dehydrogenase [Corynebacterium glutamicum ATCC 13032]. The optimized sequence of *ksaD* for Weimberg pathway construction.

ATGATCACCGCCACCGCCCTCCACGGCTGCTCCCTCATCGACGGCGAGTGGGTCGCCGGCAAGAACGGCGAGATCACCGGCTTCGACCCCCGCACCAACGCCTCCCTCAACCCCTCTTACAGTCTGGCCAACTCCGCTCAGCTCCGCGCGGCCACCACATCCGCCAAGCGCGCCTTCGAGTCCTACCGCCTCACCACCCCCGAGGTCCGCGCCGACTTCCTCGACTCCATCGCCGACAATATCGATGCCCTGTCTGGCGAAATTGTGCAGCGCGCTAGTCTGGAGACCGGCCTCGGGACCACCCGCCTTACCGGCGAGGTCGCCCGCACTAGCAACCAATTGCGGCTGTTCGCCGAGACCGTCCGGTCCGGACAGTTTCACCGCGTCCGCATAGAGCGTGGCCCGCGCATCGATTTACGCCAACGTCAAGTCCCTCTCGGCCCCGTTGCCGTCTTCGGCGCGTCCAACTTTCCCGTCGCATTCTCCACCGCCGGCGGAGATACCGCCTCCGCCCTTGCCGCCGGATGTCCCGTAGTCTTCAAGGCTCACAACGCACATCCCGGCACCGCTGAGCTCGTCGGACAAGCCGTTCGCGGCGCCGTCGAGAAGCACGAGTTCGACGCCGGCGTCTTCAACCTCGTCTACGGCCGCGGCGTCGAGATCGGCCAAGAGCTCGCCGCCGACCCCAACATCACCGCCATCGGCTTCACCGGCTCCCGTCAAGGCGGCCTGGCCCTCTCGCAAACCGCCTTCTCCCGCCCTGTCCCCGTCCCCGTCTTCGCCGAGATGTCCGCCACAAACCCGGTCTTCGTATTCCCCGGCGCCCTCGCCGATCTCGACGCTTCCTCCTCCCTCGCTGAGGCTTTCACCGCTTCCGTCACCGGATCTTCGGGTCAGCTATGCACGAAACCGGGCCTTGTCTTCATCCCACGCGGCGTCGTAGGCGACGCCTTCGTCGCCCTCGTCGCCGCCAAGTTCAAGGAGACCACCGGTCAGACCATGCTGACGCAAGGCATAGCCCAAGCATGGCAGCGCGGTGTCGACAACTTAGCTGCTCAGCCATCCGTAAAAATCCTCGCACAAGGCACCCCCGGTGACGGCGAGAATGCCCCTGGGCCCGTCGTCTTCGAGAGCGACGTCCAAGCCCTCCTCAACAACGTCGTCCTCCAAGAGGAAATCTTCGGCGCCGCCTCCCTCGTCGTCCGCTACGACTCCCCCGATCAGCTCCACCAAGTCGCCAACTCCCTGGAGGGTCAGCTCACCGCCACCATCCACGCCTCCCAAGACGACTTCCAAGAGGTCTCGAAGCTCATCCCGCTGCTGGAGGACCTCGCCGGCCGGGTCCTCTACGGGGGATGGCCCACCGGCGTCGAGGTGGGCCACACGGTAATCCACGGCGGACCCTACCCCGCCACCTCCAATGCTCAGTCCACCTCCGTCGGCACCCTCGCCATCGAGCGCTTCATGCGCCCGGTCAGCTACCAAACCTTCCCCGCCGAATTGCTCCCTGACCCCGTCTCCGAGGCCAACAAGTGGGCCGTCCCCCGCGAGATCGACCGCTAA

>WP_010918705.1 SMP-30/gluconolactonase/LRE family protein [Caulobacter vibrioides]. The optimized sequence of *xylC* for engineering *M. thermophila* to produce 1,2,4‐butanetriol.

atgactgctcaggtcacctgtgtttgggacttgaaggccactttgggtgaaggtccaatctggcatggtgacaccttgtggttcgttgacattaaacagagaaagatccacaactaccaccctgctaccggtgaaagattctccttcgatgctccagaccaggtcacctttttggctcctattgtcggtgctaccggtttcgtcgttggtttgaagaccggtattcaccgtttccacccagccactggtttctccttgttgttggaggttgaggacgctgccttgaacaacagacctaacgatgccaccgtcgatgctcaaggtagattgtggttcggcactatgcacgacggtgaggagaacaactccggttccttgtaccgtatggacttgaccggtgttgccagaatggacagagacatctgcatcactaacggaccttgcgtttccccagacggtaagaccttttaccacaccgatactttggagaagaccatttacgctttcgacttggccgaggacggattgttgtccaacaagcgtgtcttcgtccaattcgccttgggtgacgacgtctacccagacggttctgttgttgattccgagggttacttgtggaccgctttgtggggtggtttcggtgccgttagattctctccacagggtgacgctgttaccagaattgagttgccagccccaaacgtcactaagccttgtttcggtggtcctgatttaaagaccttgtacttcaccaccgctcgtaagggtttgtccgatgagactttggcccaatacccattggccggtggtgtctttgccgttccagttgacgttgctggtcaaccacagcacgaagtcagattggttggatccgatggtggcttggtggctgaaggaggagaaatggcggagcggacagtccggtgtcggctgcggtga

>NP_014555.1 alcohol dehydrogenase ADH1 [Saccharomyces cerevisiae S288C]. The optimized sequence of *adh1* for engineering *M. thermophila* to produce 1,2,4‐butanetriol.

Atgtctatcccagaaactcaaaaaggtgttatcttctacgaatcccacggtaagttggaatacaaagatattccagttccaaagccaaaggccaacgaattgttgatcaacgttaaatactctggtgtctgtcacactgacttgcacgcttggcacggtgactggccattgccagttaagctaccattagtcggtggtcacgaaggtgccggtgtcgttgtcggcatgggtgaaaacgttaagggctggaagatcggtgactacgccggtatcaaatggttgaacggttcttgtatggcctgtgaatactgtgaattgggtaacgaatccaactgtcctcacgctgacttgtctggttacacccacgacggttctttccaacaatacgctaccgctgacgctgttcaagccgctcacattcctcaaggtaccgacttggcccaagtcgcccccatcttgtgtgctggtatcaccgtctacaaggctttgaagtctgctaacttgatggccggtcactgggttgctatctccggtgctgctggtggtctaggttctttggctgttcaatacgccaaggctatgggttacagagtcttgggtattgacggtggtgaaggtaaggaagaattattcagatccatcggtggtgaagtcttcattgacttcactaaggaaaaggacattgtcggtgctgttctaaaggccactgacggtggtgctcacggtgtcatcaacgtttccgtttccgaagccgctattgaagcttctaccagatacgttagagctaacggtaccaccgttttggtcggtatgccagctggtgccaagtgttgttctgatgtcttcaaccaagtcgtcaagtccatctctattgttggttcttacgtcggtaacagagctgacaccagagaagctttggacttcttcgccagaggtttggtcaagtctccaatcaaggttgtcggcttgtctaccttgccagaaatttacgaaaagatggaaaagggtcaaatcgttggtagatacgttgttgacacttctaaataa

>ACL94327.1 xylonate dehydratase *xylD* [Caulobacter vibrioides NA1000]. The optimized sequence of *xylD* for engineering *M. thermophila* to produce 1,2,4‐butanetriol.

atgagatccgctttgtctaacagaactcctcgtcgtttcagatcccgtgactggttcgacaacccagaccatatcgacatgactgctttgtacttggaacgttttatgaactacggtattacccctgaagaacttcgttctggtaaacctatcatcggaatcgcccagaccggttctgacatttctccatgtaaccgtattcatttggatttggtccagagagtcagagacggtattcgtgacgctggtggtatcccaatggagttccctgtccaccctattttcgagaactgtcgtcgtccaaccgctgctttggacagaaacttgtcttatttgggtttggttgagaccttgcacggttacccaatcgacgctgttgtcttgactaccggttgtgacaagactaccccagctggtatcatggctgccaccactgttaacatccctgccattgtcttgtccggtggtcctatgcttgatggttggcacgagaacgagttggtcggttccggcactgttatttggagatcccgtcgtaaattggccgccggtgaaattaccgaggaggagttcatcgacagagctgcttcctctgccccatctgctggtcattgcaacactatgggcactgcctccactatgaacgctgttgctgaggctttgggactttctttgaccggttgtgctgctatccctgctccatatagagagagaggtcagatggcttacaagaccggtcagcgtatcgttgaccttgcttacgatgatgttaagccattggacattttgaccaagcaggcctttgagaacgctattgccttggtcgctgctgccggtggttctaccaatgctcaaccacatattgttgctatggcccgtcacgctggtgtcgagattactgccgatgactggagagccgcctacgatattccacttatcgtcaacatgcaaccagccggaaagtatcttggtgagagattccatagagccggaggtgctcctgccgttttgtgggagttgttgcagcagggtagattgcatggagacgtcttgactgtcactggaaagactatgtctgaaaatttgcaaggaagagagacttccgaccgtgaggtcatcttcccataccatgaaccattggccgagaaggccggtttccttgtcttgaagggtaacttgttcgattttgccatcatgaaatcctctgttattggtgaggagtttcgtaagcgttacttgtcccagccaggtcaagaaggtgtctttgaggctagagccatcgttttcgacggttctgacgactaccacaagcgtatcaatgaccctgccttggagatcgacgagagatgtattttggtcatccgtggtgctggacctattggatggccaggttctgctgaggtcgtcaatatgcaacctccagaccatttgcttaagaagggaattatgtccttgccaactttgggtgacggtagacaatctggcactgccgattccccatctatcttgaacgcctctcctgagtccgctattggaggtggtttgtcttggttgagaactggtgacaccatcagaatcgatttgaacactggaagatgcgacgccttggttgatgaggctaccattgctgccagaaagcaggacggtattccagctgttccagccaccatgactccttggcaagaaatctaccgtgcccacgcttcccaattggatactggtggagtcttggagttcgccgttaagtaccaggatttggccgccaaacttcctagacacaatcattaa

> QII57472.1, *Lactococcus lactis*, keto-acid decarboxylase. The optimized sequence of *kviD* for engineering *M. thermophila* to produce 1,2,4‐butanetriol.

atggaggaaaatcccggaccaatgtacactgtcggtgactacttgttggaccgtttgcacgagttgggaattgaagaaatcttcggtgtccctggtgactacaacttgcagttcttggatcaaattatctctcataaggatatgaaatgggttggtaatgctaacgagttgaatgcttcttacatggccgacggttacgctcgtaccaagaaggccgccgccttccttaccacctttggtgtcggtgagttgtccgctgtcaacggtttggctggttcctacgctgaaaacttgccagtcgtcgagatcgttggttctcctacttccaaggtccagaacgagggaaagtttgttcaccacaccttggccgatggtgatttcaagcacttcatgaaaatgcacgaacctgtcaccgctgctagaaccttgttgactgccgagaacgccactgttgagatcgaccgtgttttgtccgcccttttgaaggagcgtaagcctgtctacatcaacttgcctgttgacgtcgccgccgctaaagccgaaaaaccttccttgccattgaagaaggagaactccacctccaacacctctgaccaagaaattttaaacaagattcaagagtccttgaagaatgctaagaagcctatcgtcatcaccggacacgaaatcatttctttcggtttggaaaaaactgttactcaatttatctctaagaccaagttgcctattaccactttgaattttggtaaatcttccgttgacgaggccttgccatcttttttgggtatctacaacggaactctttccgagccaaacttgaaggagttcgtcgaatccgccgacttcatcttgatgttgggtgttaagttgaccgactcttctaccggtgctttcacccaccacttgaatgagaataagatgatttctttgaacattgatgaaggaaaaatcttcaacgaacgtattcaaaacttcgattttgaatctttgatctcttcccttttggacttgtctgaaattgagtataagggtaagtatattgacaaaaaacaagaagatttcgtcccttccaacgccttgttgtcccaggatagattgtggcaggctgtcgagaacttgacccagtccaatgagaccatcgtcgctgagcaaggaacctctttcttcggtgcctcttccatttttttgaagtccaagtcccactttatcggtcagccattgtggggttctattggttacactttccctgccgctttgggatctcagatcgctgataaggagtcccgtcatcttttgttcattggtgacggttccttgcagttgaccgtccaagagttgggattggctattagagaaaagattaaccctatttgttttattattaataacgacggatacactgtcgagcgtgagattcacggacctaaccaatcttacaacgacattccaatgtggaactactccaagttgccagagtccttcggtgccactgaggacagagttgtttccaagattgttagaactgagaatgagtttgtttctgtcatgaaggaggctcaagctgacccaaaccgtatgtactggatcgagcttatcttggccaaggaaggtgccccaaaggttttgaaaaaaatgggtaagttgtttgctgaacaaaacaagtctggatccgtcaagccggcaggctttcatgcaagctttggggctacgagtcgggcggcattgggtttgcgtttgatgcatcttggttacggcgtgtatgtcatttga

>WP_016501746.1 benzoylformate decarboxylase [Pseudomonas putida]. The optimized sequence of *mdlC* for engineering *M. thermophila* to produce 1,2,4‐butanetriol.

ATGGCCAGCGTCCACGGCACCACCTACGAATTATTACGGCGCCAAGGTATCGACACCGTGTTCGGTAACCCCGGCAGCAACGAGCTCCCGTTCCTCAAGGACTTCCCGGAGGACTTCCGCTACATTTTAGCCCTCCAAGAAGCTTGTGTGGTCGGCATCGCCGATGGGTACGCCCAAGCCAGCCGCAAGCCGGCGTTCATCAATTTACATTCGGCGGCCGGCACCGGCAACGCGATGGGCGCTTTAAGCAACGCTTGGAACTCGCACAGCCCTTTAATTGTCACCGCCGGCCAACAGACGCGGGCGATGATTGGCGTCGAGGCGCTGCTCACCAACGTCGACGCCGCCAATTTACCCCGGCCCCTCGTCAAGTGGAGCTACGAGCCGGCGAGCGCCGCGGAGGTCCCTCATGCGATGAGCCGGGCGATCCATATGGCGAGCATGGCCCCTCAAGGCCCCGTCTATTTATCGGTCCCGTACGACGACTGGGACAAGGACGCCGACCCTCAGTCGCACCACCTCTTCGACCGCCACGTGAGCTCGTCGGTCCGGCTGAACGACCAAGATCTCGATATTCTCGTCAAGGCTTTAAACAGCGCCTCGAATCCCGCCATTGTTTTAGGCCCCGACGTCGATGCCGCCAATGCCAACGCGGACTGTGTGATGCTGGCCGAGCGTTTAAAGGCCCCGGTGTGGGTCGCCCCGAGCGCCCCGCGCTGCCCCTTTCCGACCCGCCATCCGTGCTTTCGGGGCCTCATGCCCGCCGGCATTGCCGCCATCTCGCAGTTATTAGAAGGCCACGATGTGGTTTTAGTGATCGGGGCCCCTGTGTTTCGGTATCACCAGTACGACCCGGGCCAGTATCTCAAGCCGGGGACCCGCCTCATTAGCGTCACTTGTGACCCGCTCGAAGCCGCCCGCGCCCCGATGGGTGATGCCATCGTCGCCGATATTGGGGCCATGGCCTCGGCGCTCGCCAATCTCGTCGAGGAATCGAGCCGCCAGCTCCCGACCGCCGCCCCTGAGCCGGCCAAAGTCGATCAAGATGCGGGCCGCCTCCACCCGGAGACGGTGTTCGATACCCTCAACGACATGGCCCCTGAGAACGCCATCTATTTAAACGAGTCGACCTCGACCACCGCCCAGATGTGGCAGCGGCTGAACATGCGCAACCCCGGTAGCTACTACTTTTGCGCCGCCGGCGGTTTAGGCTTTGCCCTCCCGGCCGCCATCGGCGTCCAACTCGCGGAACCGGAACGCCAAGTTATCGCCGTCATCGGCGACGGGTCGGCCAACTACTCGATCAGCGCCCTCTGGACCGCCGCCCAGTACAACATCCCCACCATCTTCGTCATCATGAACAACGGCACCTACGGGGCCCTCCGCTGGTTCGCCGGGGTGCTCGAGGCCGAAAACGTCCCGGGGCTCGATGTCCCCGGCATCGACTTCCGCGCTTTAGCGAAAGGCTACGGCGTCCAAGCCCTCAAAGCCGACAACCTCGAGCAGCTGAAAGGCTCGCTGCAAGAAGCCCTCAGCGCCAAAGGCCCGGTTTTAATCGAGGTCTCGACCGTGAGCCCCGTCAAGTGA
